# *RNF213* Variants Associated With Periventricular Anastomosis Regression After Revascularization in Moyamoya Disease

**DOI:** 10.1002/cns.70982

**Published:** 2026-06-12

**Authors:** Youyuan Bao, Chaofan Zeng, Yimeng Xue, Qianjun Zhao, Xudong Sun, Fanbo Meng, Haoyuan Chen, Yunhao Cui, Qi Yuan, Hao Li, Dong Zhang, Rong Wang, Yan Zhang, Jizong Zhao, Qian Zhang

**Affiliations:** ^1^ Department of Neurosurgery, Beijing Tiantan Hospital Capital Medical University Beijing China; ^2^ China National Clinical Research Center for Neurological Diseases Beijing China; ^3^ Center of Stroke Beijing Institute for Brain Disorders Beijing China; ^4^ Beijing Key Laboratory of Translational Medicine for Cerebrovascular Disease Beijing China; ^5^ Department of Neuropathology Beijing Neurosurgical Institute Beijing China; ^6^ Capital Medical University Beijing China; ^7^ Department of Neurosurgery Beijing Hospital Beijing China; ^8^ Institute of Geriatric Medicine Chinese Academy of Medical Sciences Beijing China

**Keywords:** cerebral revascularization, intracranial hemorrhage, moyamoya disease, periventricular anastomosis, *RNF213*

## Abstract

**Aims:**

This study aimed to investigate the characteristics, predictors, and clinical significance of periventricular anastomosis (PA) regression following revascularization in moyamoya disease (MMD).

**Methods:**

We conducted a longitudinal cohort study involving patients with MMD treated at our hospital between 2019 and 2023. Demographic, radiological findings, and clinical outcomes were recorded. C‐terminal region of ring finger protein 213 (*RNF213*) was sequenced. Additionally, structural changes of the *RNF213* variants were evaluated using a three‐dimensional model of the human RNF213 protein.

**Results:**

Of 305 eligible patients, 103 experienced postoperative PA regression. Among the subtypes of PA, those derived from the lenticulostriate, anterior choroidal, and thalamotuberal arteries exhibited significant regression. Multivariate analysis identified age (*p* = 0.030), Suzuki stage (III‐IV) (*p* = 0.043), preoperative PA score (*p* < 0.001), *RNF213* p.R4810K variant (*p* = 0.019), other *RNF213* rare variants (*p* = 0.004), and postoperative Matsushima grading (*p* < 0.001) were associated with postoperative PA regression. The risk of future hemorrhage was significantly higher in the non‐PA regression group (*p* = 0.040). 80% of rare variants without structural changes did not exhibit PA regression.

**Conclusions:**

*RNF213* variants in the C‐terminal region are associated with PA regression, underscoring a broader genotype–phenotype correlation. Furthermore, PA regression might be considered a potential radiological marker to predict the risk of future hemorrhage in MMD patients.

## Graphical abstract

Bao et al. demonstrated that *RNF213* variants in the C‐terminal region are associated with periventricular anastomosis (PA) regression, underscoring a broader genotype and angiographic phenotype correlation. Furthermore, PA regression could serve as a potential radiological marker for predicting the risk of future hemorrhage in moyamoya disease patients.

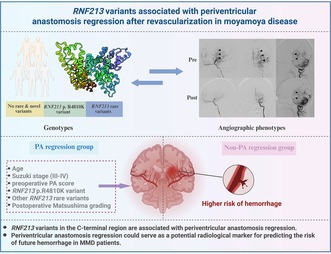

## 1 Introduction

Moyamoya disease (MMD) is a rare cerebrovascular disorder characterized by the progressive occlusion of intracranial arteries and the development of fragile, abnormal collateral vessels at the base of the brain [1]. The two primary clinical manifestations of MMD are intracranial ischemia and hemorrhage. Although intracranial hemorrhage occurs less frequently than ischemic events, it remains the leading cause of death in MMD patients [1, 2]. Therefore, the primary treatment strategy is hemorrhagic prevention through bypass surgeries.

A novel term, periventricular anastomosis (PA), has been introduced to describe the fragile nature of abnormal collateral vessels in the periventricular region [3]. According to a subsequent series from the Japan Adult Moyamoya (JAM) Trial [4, 5, 6], these abnormal collateral anastomoses have been associated with an increased risk of bleeding in MMD. Notably, a few small series have reported that PA can be normalized through revascularization procedures [7, 8, 9, 10, 11]. Consequently, it is reasonable to hypothesize that PA regression is associated with a decreased risk of hemorrhage. However, this hypothesis remains unproven in large cohort studies with long‐term follow‐up, and comprehensive investigations into the characteristics of postoperative angiographic changes in individual collateral vessels, as well as the factors contributing to such changes, remain limited. Furthermore, despite the overall success of direct bypass (DB) in reducing the risk of hemorrhage, the JAM Trial reported that approximately 12% of patients experienced rebleeding within 5 years [12]. The underlying causes of rebleeding in these patients have not yet been thoroughly explored, and the impact of bypass surgeries on these abnormal collateral anastomoses remains unclear.

In recent years, ring finger protein 213 (*RNF213*) has been identified as the primary susceptibility gene for MMD in East Asian populations [13, 14]. Previous studies have demonstrated a genotype–phenotype correlation for the *RNF213* p.R4810K variant, including associations with ischemic events [15], earlier onset [15, 16], posterior cerebral artery (PCA) involvement [15, 16], and postoperative collateral formation (PCF) [16, 17]. Also, some reports have suggested that *RNF213* rare variants (RVs) are linked to clinical phenotypes in MMD patients across ethnically diverse populations [18, 19, 20, 21]. Recently, our team was the first to confirm that *RNF213* p.R4810K and other RVs in the C‐terminal region are also associated with PA in MMD [22]. Nevertheless, the role of *RNF213* variants in postoperative PA regression remains unclear. We hypothesize that MMD patients carrying the *RNF213* p.R4810K or C‐terminal RVs are more likely to exhibit PA regression than those without these variants.

Based on these observations, this study aimed to: (1) determining the impact of revascularization surgeries on PA within a large MMD cohort; (2) analyze the characteristics of postoperative angiographic changes in each PA subtype; (3) identify factors associated with postoperative PA regression and explore whether PA regression can reduce future hemorrhage risk. Additionally, to better characterize the relationship between genotype and PA phenotype, the structural changes of the *RNF213* variants in this series were evaluated using a three‐dimensional (3D) model of the human RNF213 protein.

## 2 Methods

### 2.1 Study Population and Design

The study was approved by the institutional review board and ethics committee of the Beijing Tiantan Hospital, and written informed consent was obtained from all patients or their guardians. It was conducted in accordance with the Strengthening the Reporting of Observational Studies in Epidemiology (STROBE) guidelines and the 2024 revision of the Declaration of Helsinki.

A consecutive series of patients with MMD who visited Beijing Tiantan Hospital, Capital Medical University, between June 2019 and January 2021 were prospectively enrolled in the original study (NCT04906564). From July 2021 to November 2023, we continued to prospectively collect data from MMD patients in the single‐diagnosis group, along with follow‐up assessments for all participants. The inclusion criteria were as follows: (1) diagnosis of MMD based on digital subtraction angiography (DSA) according to the current diagnostic criteria [23]; (2) received surgical revascularization, including indirect bypass (IB) and combined bypass (CB); (3) had complete DSA performed before and 6 ± 3 months after surgery; (4) underwent computed tomography perfusion (CTP) examination prior to surgery; and (5) had sequencing of exons in the C‐terminal region of the *RNF213* gene. Patients with Moyamoya syndrome due to other systemic diseases or conditions interfering with study outcomes were excluded. Given the potential for contralateral cerebral hemodynamic changes following unilateral revascularization [24], only the surgical hemisphere (the hemisphere receiving the initial surgery) was included in the primary analysis. The nonsurgical hemisphere (the hemisphere receiving surgery later) was used for intra‐individual comparison of PA scores.

### 2.2 Clinical Data Collection

Clinical information for the included patients was obtained from the electronic medical record system. Data on demographics, risk factor history, clinical presentation, modified Rankin Scale (mRS) scores, surgical modalities, and follow‐up details were prospectively collected at the onset of the study.

Two types of surgical modalities were analyzed: CB and IB. CB involves the integration of direct bypass (DB) with either encephaloduroarteriosynangiosis (EDAS) or encephalodurosynangiosis, while IB includes EDAS and/or multiple burr hole drilling. The detailed procedures and revascularization principles have been described previously [25].

### 2.3 DNA Sequencing for *RNF213* Variants

Genomic DNA was extracted from peripheral blood samples of each participant using the QIAamp DNA Blood Kit (Qiagen, Hilden, Germany). Mutational analysis of the C‐terminal region of *RNF213* (NM_001256071, exons 42–68) was conducted through direct Sanger sequencing for all cases. Polymerase chain reaction (PCR) was carried out with the Thermal Cycler 2720 (Applied Biosystems, Foster City, CA, USA), followed by sequencing and analysis of the PCR products using an Applied Biosystems 3130xl Genetic Analyzer (Thermo Fisher Scientific). The primer sequences were identical to those used in our previous study [15]. Allele frequencies for each variant were analyzed using the Genome Aggregation Database (gnomAD) (v.2.1.1), with RVs defined as having a minor allele frequency of < 0.001 in gnomAD. The Combined Annotation‐Dependent Depletion (CADD; GRCh37‐v1.6) algorithms were employed to evaluate the functional effects of variants [26]. To predict the potential impact of amino acid changes, the Rare Exome Variant Ensemble Learner (REVEL) score and PolyPhen‐2 were applied. All data analyses were conducted at the Department of Neurosurgery, Beijing Tiantan Hospital.

### 2.4 Radiological Evaluation and Classification

Preoperative vasculature characteristics were evaluated with DSA by two independent neurosurgeons who were unaware of the clinical details. Any disagreements were resolved by consensus. The Suzuki stage was used to evaluate each hemisphere, grading it into six distinct stages. PCA involvement was defined as occlusion or stenosis in segments P1 to P3 [22]. PA was classified as lenticulostriate artery (LSA), thalamotuberal artery (TTA), thalamoperforating artery (TPA), anterior choroidal artery (AChoA), and posterior choroidal artery (PChoA) based on where the vessels run [3]. The degree of PA dilation was assessed using the criteria proposed by Morioka et al. [27], with minor modifications, and categorized into three grades: Grade 0, where the perforating arteries show no dilation or extension; Grade 1, where dilated or extended arteries reach the level of the lateral ventricle; and Grade 2, where dilated or extended arteries extend beyond the level of the lateral ventricle. Each PA vessel was scored as 0 point for “Grade 0/1” and 1 point for “Grade 2,” with the sum of these subtype scores representing the PA score for each patient, ranging from 0 to 5. Collateral circulation was assessed using the classification criteria established by Liu et al. [28] Any collateral vessels arising from a middle meningeal artery, superficial temporal artery, or occipital artery were defined as the presence of spontaneous external carotid artery (ECA) compensation. Additionally, cerebral hemodynamic status was evaluated with CTP. The stages of the preinfarction period were determined according to the criteria defined in our previous study [29].

Angiographic follow‐up was typically scheduled 6 ± 3 months postoperatively to assess disease progression and the effectiveness of revascularization. The formation of new collateral vessels from the ECA system following revascularization was graded using the Matsushima grade [30]. According to this scale, Grades A and B were classified as “good” PCF, while Grades C were classified as “poor” PCF (Figure S1). As a primary variable of interest, changes in PA were evaluated by comparing preoperative and postoperative angiograms. Specifically, PA regression was defined as any decrease in the PA subtype grade, such as from Grade 2 to 1 or 0 or from Grade 1 to 0. Conversely, PA progression was defined as an increase in the PA subtype grade, such as from Grade 0 to 1 or 2, or from Grade 1 to 2. If the grade remains unchanged, it was classified as no change. PA regression was then recorded as a dichotomous variable (yes/no). Representative angiograms of PA regression are shown in Figure S2.

### 2.5 Clinical Follow‐Up

After discharge, follow‐up evaluations were conducted through clinic visits or telephone interviews 3–6 months after surgery and annually thereafter. To better capture long‐term stroke events, patients lost to follow‐up or had a follow‐up duration shorter than one year were excluded from the study. The main outcome was recurrent cerebrovascular events, which encompasses intracranial hemorrhage, cerebral infarction, and transient ischemic attack (TIA). Only events occurring in the hemisphere of interest were included in the analysis. Follow‐up hemorrhagic or ischemic stroke was defined as new neurological deficits lasting more than 24 h, accompanied by newly identified hemorrhage or infarct on CT scan and/or diffusion‐weighted MR. TIA was defined as a transient neurological deficit lasting less than 24 h without radiographic evidence of stroke.

### 2.6 Molecular Modeling of Human RNF213

To model the 3D structure of the *RNF213* p.R4810K variant and other RVs identified in the study, we used the coordinate data of the mouse RNF213 3D structure (UniProt: E9Q555, PDB ID: 6TAX) as a template [31], sourced from the Protein Data Bank (http://www.rcsb.org/pdb/). A 3D model of human RNF213 was generated through homology modeling using AlphaFold3, and mutant variant models were constructed using ChimeraX (v1.4). The steric configurations of the wild‐type and variant proteins were then compared and visualized using PyMOL (v3.0).

### 2.7 Statistical Analysis

Statistical analyses were conducted using R Statistical Software (version 4.3.2, R Foundation for Statistical Computing). Clinical data were presented as percentages, means, or medians with interquartile ranges, depending on the variable type. Continuous variables were compared using the Student *t*‐test or the Mann–Whitney U‐test, while categorical variables were assessed using the chi‐square test or Fisher's exact test, as appropriate. Interrater agreement was assessed using the intraclass correlation coefficient for the overall PA score and weighted kappa statistics for the grading of each PA subtype. Changes in PA scores before and after surgery were evaluated using the Wilcoxon signed‐rank test. Kaplan–Meier methods were employed to generate cumulative stroke incidence curves, with group comparisons conducted using the log‐rank test.

To identify predictors of postoperative PA regression, variables with a *p* value < 0.05 in univariate logistic regression analysis were included in the multivariable analysis. The potential confounder (sex and preinfarction period stage) was adjusted in the multivariate regression model based on clinical relevance. The variance inflation factor (VIF) was calculated to detect multicollinearity among the variables included in the final multivariable logistic regression model. A VIF of > 5 indicates a high correlation of the variables. No missing data were observed for the key variables included in the primary analyses; therefore, no imputation was required. For all analyses, *p* < 0.05 was considered statistically significant.

## 3 Results

### 3.1 Patient Demographics and Clinical Characteristics

**FIGURE 1:**
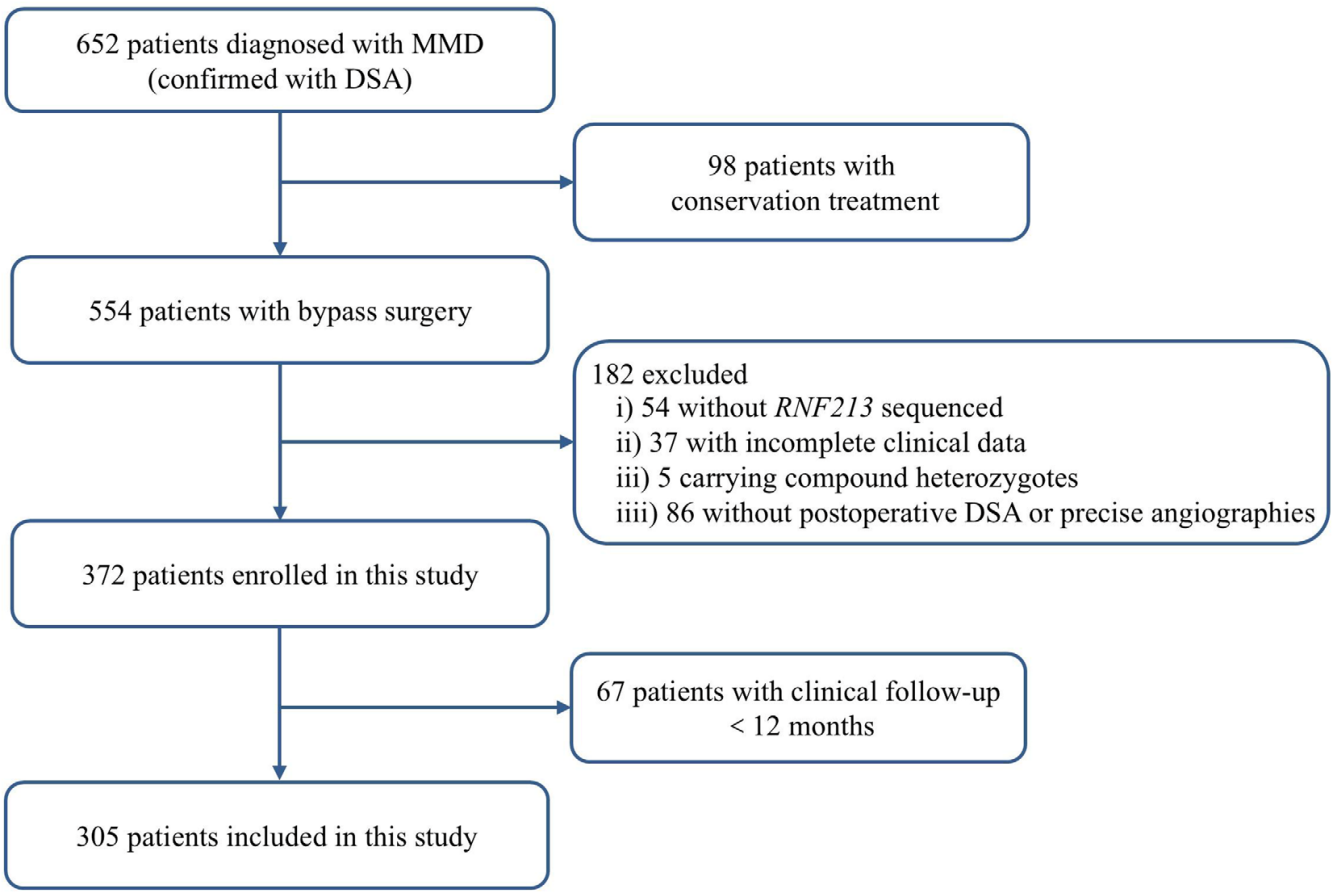
Flowchart of participants in this study. The flow diagram illustrated the inclusion and exclusion process of moyamoya disease (MMD) for the study. DSA, digital subtraction angiography.

A total of 652 patients with MMD were identified at our center. Of these, 305 patients were ultimately included in the study (Figure 1). Female patients comprised 54.1% of the cohort, and the median age was 35.0 years (interquartile range [IQR] 15.0–46.0). Among the 305 hemispheres that underwent initial revascularization, 103 (33.8%) exhibited postoperative PA regression, compared to only 10 of 305 nonsurgical hemispheres (3.3%). The difference between the surgical and nonsurgical hemispheres was statistically significant (Table S1, *p* < 0.001). A significant decrease in PA scores was observed in the surgical hemisphere compared to baseline (*p* < 0.001), whereas no significant difference was detected in the nonsurgical hemisphere (*p* = 0.570) (Figure S3). Additionally, interobserver agreements were reliable for the overall PA score and across individual PA subtypes, both preoperatively and postoperatively (Table S2).

The differences in patient characteristics between the PA regression and non‐PA regression groups in the surgical hemisphere were detailed in Table 1. Patients with postoperative PA regression had a younger onset age (*p* < 0.001) and exhibited more ischemic manifestations (*p* = 0.011). Significant differences in radiological features were observed, including Suzuki stage, collateral circulation, PCA involvement, preinfarction period stage, PA score, and ECA collateral between the two groups. Patients with postoperative PA regression had a higher prevalence of PCA involvement (*p* < 0.001), ECA collateral (*p* = 0.004), and higher PA scores (*p* < 0.001). In the PA regression group, 75.7% of patients underwent IB, significantly higher than the 47.5% in the non‐PA regression group (*p* < 0.001). The proportions of *RNF213* genotypes also differed significantly between the groups. Furthermore, good PCF was more frequent in the PA regression group than in the non‐PA regression group (96.1% vs. 67.3%, *p* < 0.001). No significant differences were found in other clinical characteristics, including sex, personal history, mRS score on admission, surgical side, and follow‐up time.

**TABLE 1:** Baseline demographic and clinical characteristics of the entire cohort.

Variables	Total cohort (*n* = 305)	Postoperative PA regression		*p*
		Present (*n* = 103)	Absent (*n* = 202)	
Age, y; median	35.0 (15.0–46.0)	26.0 (12.0–36.0)	40.0 (25.0–48.0)	**< 0.001** ^a^
Age group				**< 0.001** ^b^
Children, < 18	86 (28.2)	45 (43.7)	41 (20.3)	
Adults, ≥ 18	219 (71.8)	58 (56.3)	161 (79.7)	
Female sex	165 (54.1)	54 (52.4)	111 (55.0)	0.676^b^
Clinical manifestation^c^				**0.011** ^b^
Hemorrhage	74 (24.3)	16 (15.5)	58 (28.7)	
Ischemia	231 (75.7)	87 (84.5)	144 (71.3)	
Personal history				
Hypertension	84 (27.5)	23 (22.3)	61 (30.2)	0.146^b^
Diabetes	34 (11.2)	10 (9.7)	24 (11.9)	0.569^b^
Hyperlipidemia	42 (13.8)	10 (9.7)	32 (15.8)	0.142^b^
Cigarette smoking	40 (13.1)	12 (11.7)	28 (13.9)	0.589^b^
Alcohol drinking	26 (8.5)	6 (5.8)	20 (9.9)	0.228^b^
Admission mRS > 2	21 (6.9)	9 (8.7)	12 (5.9)	0.602^b^
Suzuki stage				**< 0.001** ^b^
I‐II	77 (25.3)	5 (4.9)	72 (35.6)	
III‐IV	202 (66.2)	95 (92.2)	107 (53.0)	
V‐VI	26 (8.5)	3 (2.9)	23 (11.4)	
Collateral circulation				**0.009** ^b^
Grade I (1–4)	83 (27.2)	38 (36.9)	45 (22.3)	
Grade II (5–8)	211 (69.2)	64 (62.1)	147 (72.8)	
Grade III (9–12)	11 (3.6)	1 (1.0)	10 (5.9)	
PCA involvement	87 (28.5)	50 (48.5)	37 (18.3)	**< 0.001** ^b^
Stage of the preinfarction period				**0.029** ^b^
1	14 (4.6)	0 (0.0)	14 (6.9)	
2	98 (32.1)	32 (31.1)	66 (32.7)	
3	133 (43.6)	52 (50.5)	81 (40.1)	
4	60 (19.7)	19 (18.4)	41 (20.3)	
Pre PA score	1.0 ± 1.3	2.0 ± 1.3	0.5 ± 0.9	**< 0.001** ^b^
ECA collateral	100 (32.8)	45 (43.7)	55 (27.2)	**0.004** ^b^
Surgery type				**< 0.001** ^b^
Combined	131 (43.0)	25 (24.3)	106 (52.5)	
Indirect	174 (57.0)	78 (75.7)	94 (47.5)	
Surgical side, lt	175 (57.9)	64 (62.1)	111 (55.0)	0.230^b^
Genotype of *RNF213*				**< 0.001** ^b^
w/o rare variants	209 (68.5)	44 (42.7)	165 (81.7)	
w p.R4810K variant	81 (26.6)	49 (47.6)	32 (15.8)	
w rare variants	15 (4.9)	10 (9.7)	5 (2.5)	
Post Matsushima grading				**< 0.001** ^b^
Good	235 (77.0)	99 (96.1)	136 (67.3)	
Poor	70 (23.0)	4 (3.9)	66 (32.7)	
Follow‐up, mos	45.9 ± 24.9	45.4 ± 27.7	46.2 ± 23.4	0.785^d^

*Note:* Values are shown as number (%), mean ± SD, or median IQR unless indicated otherwise. Boldface type indicates statistical significance (*p* < 0.05). Abbreviations: ECA, external carotid artery; mRS, modified Rankin Scale; PA, periventricular anastomosis; PCA, posterior cerebral artery.

^a^ Nonparametric tests: Mann–Whitney‐U.

^b^ Chi‐square test.

^c^ Patients with ischemia, seizure, transient ischemic attack, and headache were classified into the ischemic group.

^d^ *t*‐test.

**FIGURE 2:**
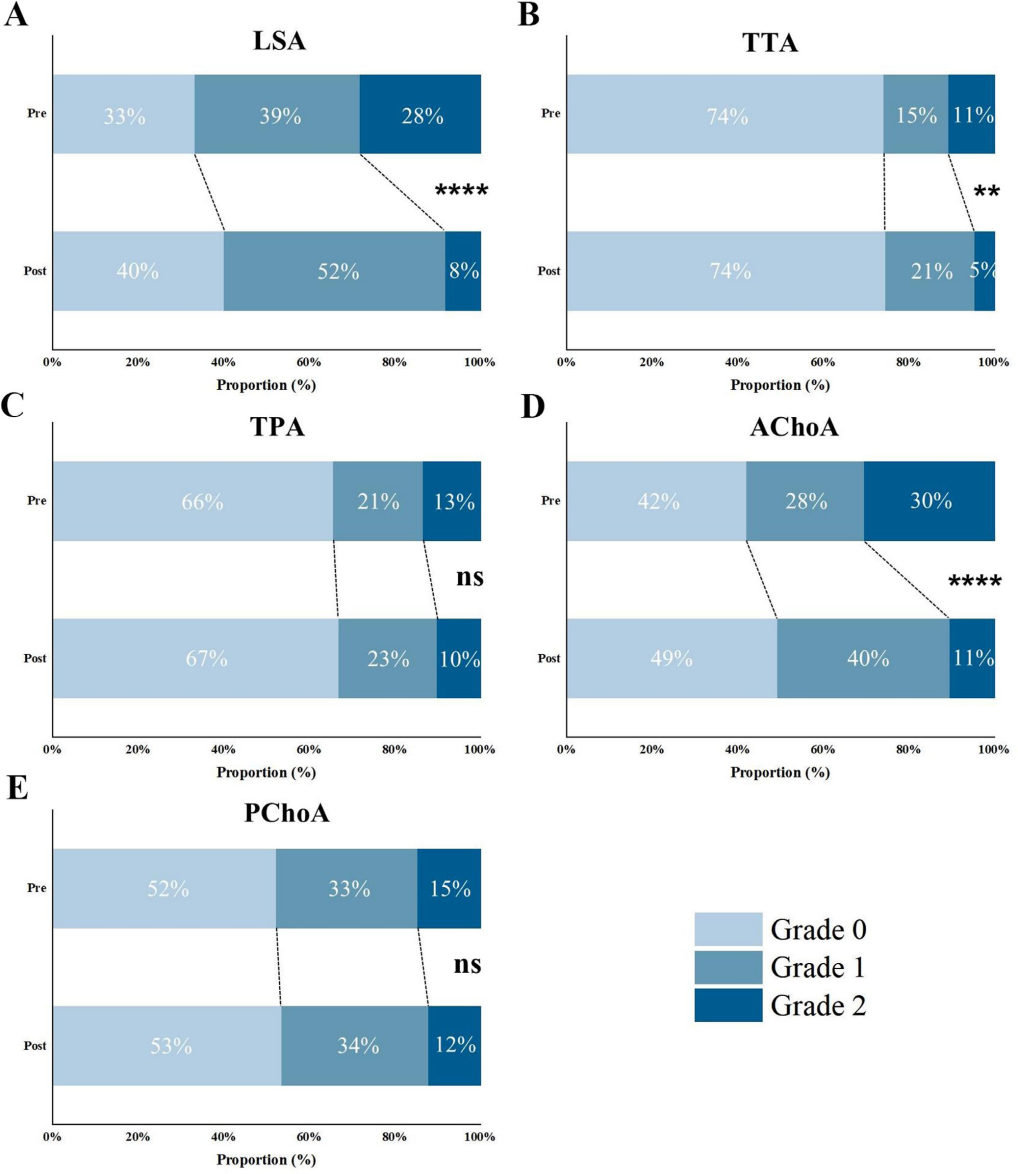
Characteristics of angiographic changes in PA subtypes before and after surgery in 305 hemispheres. (A–E) The stacked chart showed that the LSA (A), TTA (B), and AChoA (D) showed significant regression (*p* < 0.0001, *p* = 0.0049, and *p* < 0.0001, respectively), while no notable changes were observed in the TPA (C) and PChoA (E) (*p* = 0.428 and *p* = 0.635, respectively). LSA, lenticulostriate artery; AChoA, anterior choroidal artery; PChoA, posterior choroidal artery; TPA, thalamoperforating artery; TTA, thalamotuberal artery. *p < 0.05; **p < 0.01; ***p < 0.001; ****p < 0.0001.

The preoperative and postoperative changes for each PA subtype in the entire cohort were also evaluated (Figure 2). Preoperatively, dilation of the LSA was the most prominent, with more than 60% of hemispheres classified as Grade 1 or 2 (Figure 2A). After unilateral revascularization surgery, the LSA, TTA, and AChoA showed significant regression (*p* < 0.001, *p* = 0.005, and *p* < 0.001, respectively), while no notable changes occurred in the TPA and PChoA (*p* = 0.428 and *p* = 0.635, respectively). When patients were stratified into pediatric and adult groups, the results were generally similar but with some differences (Figure S4). Preoperative PA development was more pronounced in the pediatric group compared to the adult group. Additionally, TTA did not show significant postoperative changes in the adult group.

### 3.2 Predictors Associated With Postoperative PA Regression

The results of the univariable and multivariable analyses identifying predictors for postoperative PA regression are presented in Table 2. In the univariable analysis, variables related to postoperative PA regression included age at operation, clinical manifestation, Suzuki stage, collateral circulation, PCA involvement, preoperative PA score, ECA collateral, surgery type, *RNF213* variants, and PCF. After adjusting for sex and preinfarction period stage, age at operation (Odds ratio [OR] 0.974, 95% confidence interval [CI] 0.951–0.997, *p* = 0.030), Suzuki stages III‐IV (OR 3.461, 95% CI 1.040–11.522, *p* = 0.043), preoperative PA score (OR 2.124, 95% CI 1.551–2.908, *p* < 0.001), *RNF213* p.R4810K variant (OR 2.404, 95% CI 1.158–4.994, *p* = 0.019), other *RNF213* RVs (OR 8.443, 95% CI 2.003–35.598, *p* = 0.004), and PCF (OR 0.056, 95% CI 0.013–0.238, *p* < 0.001) remained significantly associated with postoperative PA regression. Multicollinearity diagnosis confirmed that the VIF of all variables was below 5 (Table S3, ranging from 1.076 to 1.544), ensuring the stability of the model estimates.

**TABLE 2:** Univariate and multivariate logistic regression analyses of factors associated with postoperative PA regression.

Variables	Univariate Analysis		Multivariate Analysis	
	OR (95% CI)	*p*	OR (95% CI)	*p*
Age, y	0.959 (0.944–0.974)	**< 0.001**	0.974 (0.951–0.997)	**0.030**
Sex, male	1.107 (0.688–1.781)	0.676	1.098 (0.563–2.143)	0.783
Clinical manifestation				
Ischemia	1.0 (Reference)		1.0 (Reference)	
Hemorrhage	0.457 (0.247–0.844)	**0.012**	0.572 (0.255–1.284)	0.176
Personal history				
Hypertension	0.665 (0.382–1.155)	0.147		
Diabetes	0.797 (0.366–1.738)	0.569		
Hyperlipidemia	0.571 (0.269–1.214)	0.145		
Cigarette smoking	0.819 (0.398–1.687)	0.589		
Alcohol drinking	0.356 (0.185–1.835)	0.356		
Admission mRS > 2	0.771 (0.290–2.051)	0.602		
Suzuki stage				
I‐II	1.0 (Reference)		1.0 (Reference)	
III‐IV	10.542 (4.383–25.358)	**< 0.001**	3.461 (1.040–11.522)	**0.043**
V‐VI	1.565 (0.362–6.762)	0.548	1.088 (0.174–6.804)	0.928
Collateral circulation				
Grade I (1–4)	1.0 (Reference)		1.0 (Reference)	
Grade II (5–8)	0.516 (0.306–0.869)	**0.013**	0.550 (0.244–1.240)	0.150
Grade III (9–12)	0.118 (0.014–0.968)	**0.047**	1.592 (0.131–19.418)	0.715
PCA involvement	4.207 (2.487–7.117)	**< 0.001**	1.387 (0.629–3.056)	0.417
Stage of the preinfarction period	1.233 (0.917–1.659)	0.166	1.085 (0.706–1.667)	0.710
Pre PA score	3.150 (2.398–4.136)	**< 0.001**	2.124 (1.551–2.908)	**< 0.001**
ECA collateral	2.074 (1.261–3.411)	**0.004**	1.687 (0.831–3.427)	0.148
Surgery type				
Indirect	1.0 (Reference)		1.0 (Reference)	
Combined	0.290 (0.171–0.492)	**< 0.001**	0.873 (0.402–1.896)	0.732
Surgical side, lt	0.743 (0.458–1.207)	0.231		
Genotype of *RNF213*				
w/o rare variants	1.0 (Reference)		1.0 (Reference)	
w p.R4810K variant	5.742 (3.293–10.012)	**< 0.001**	2.404 (1.158–4.994)	**0.019**
w rare variants	7.500 (2.438–23.075)	**< 0.001**	8.443 (2.003–35.598)	**0.004**
Post Matsushima grading				
Good	1.0 (Reference)		1.0 (Reference)	
Poor	0.083 (0.029–0.236)	**< 0.001**	0.056 (0.013–0.238)	**< 0.001**

*Note:* Boldface type indicates statistical significance (*p* < 0.05).

### 3.3 Follow‐Up Outcomes

**FIGURE 3:**
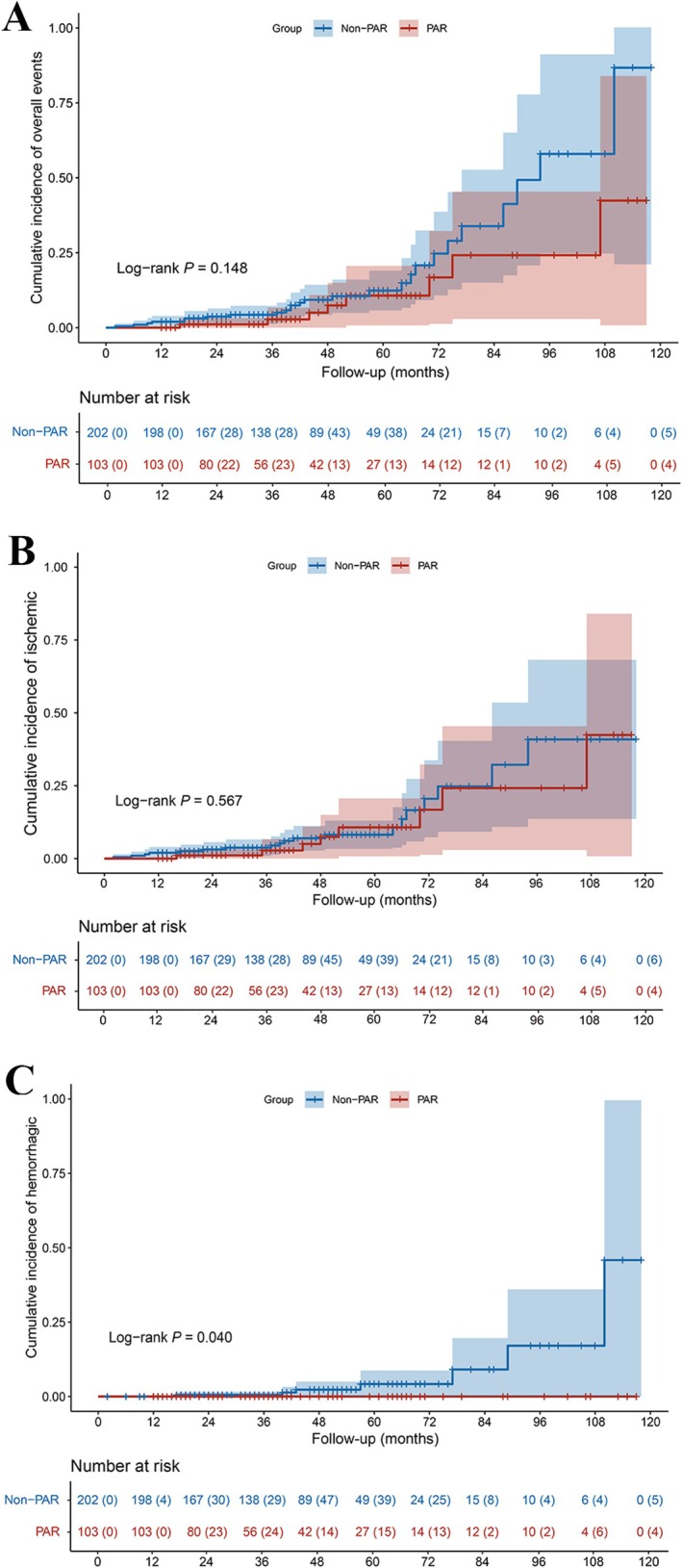
Kaplan–Meier cumulative curves for stroke recurrence in 305 patients with MMD. (A–C) Kaplan–Meier curve analysis using the log‐rank test showed the risk of all ischemic and hemorrhagic events (A), ischemic events only (B), and hemorrhagic events only (C). There was a significant difference in the rate of hemorrhage between the PA regression and non‐PA regression groups.

During a mean follow‐up of 45.9 months, recurrent cerebrovascular events occurred in 34 (11.1%) hemispheres, including 27 (8.9%) with ischemic events (22 with TIA and 5 with cerebral infarctions) and 7 (2.3%) with hemorrhagic stroke. None of the hemispheres with postoperative PA regression experienced hemorrhage, whereas 7 (3.5%) of 202 hemispheres without postoperative PA regression did. The cumulative incidence curves demonstrated a significantly higher risk of hemorrhage in the non‐PA regression group compared to the PA regression group (Figure 3C), whereas the overall incidence of stroke or ischemia did not differ significantly (Figure 3A,B).

### 3.4 DNA Sequencing Results and Structural Analysis of *RNF213* Variants

**FIGURE 4:**
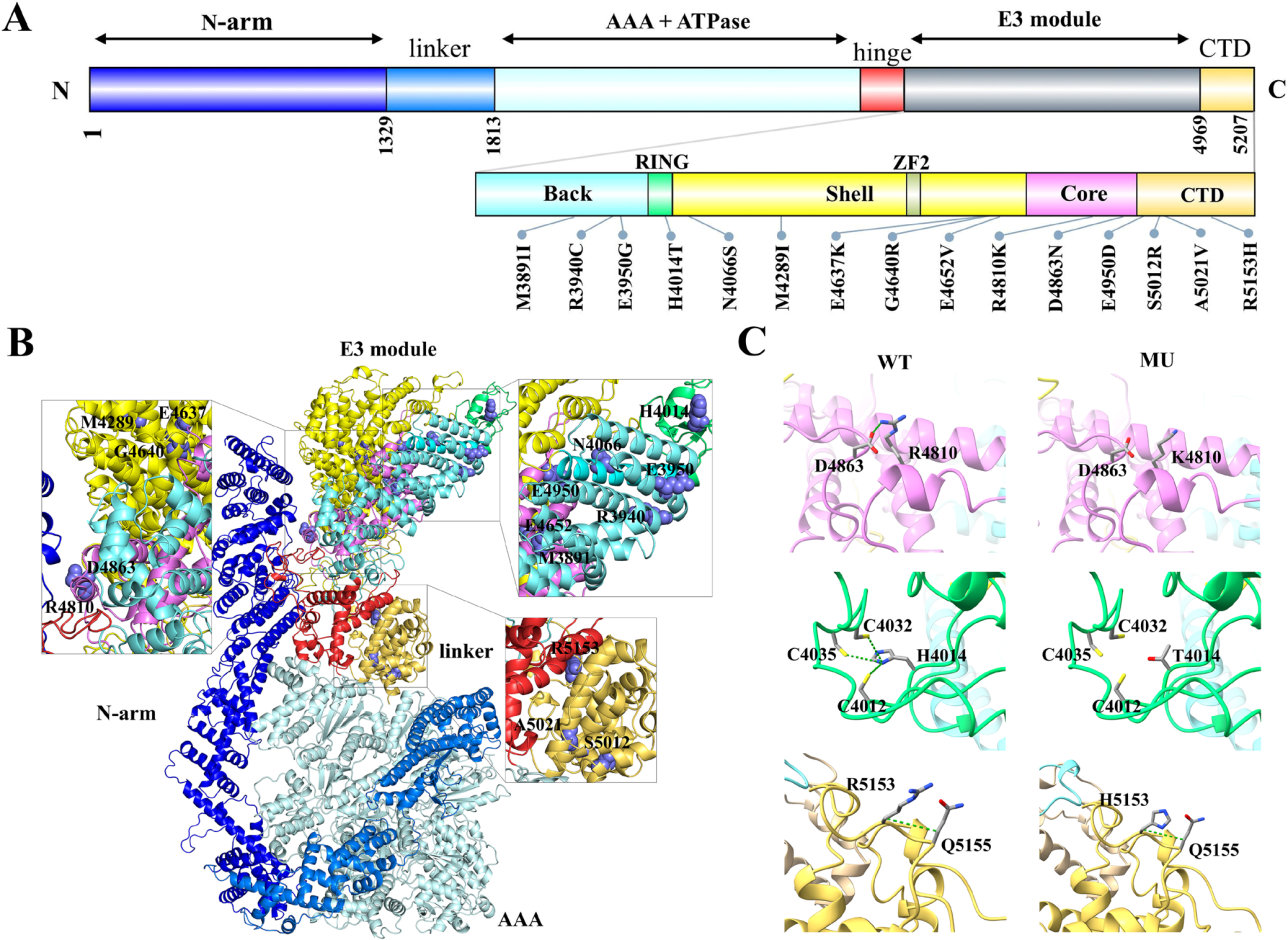
Schematic representation of the RNF213 protein and molecular modeling of Human RNF213. (A) Representation of the RNF213 protein and variants identified in our study. In the close‐up of the C‐terminal region, the 15 variants are presented in detail. (B) The diagram illustrates the model of the 3D structure of human RNF213. The *RNF213* p.R4810K variant and other C‐terminal rare variants are mapped. (C) The structural change of p.R4810K, p.H4014T, and p.R5153H. In the WT (wild type), R4810 in the E3 core domain forms a salt bridge with D4863. After MU (mutation), it is assumed that this salt bridge is lost due to the replacement of R4810 with K4810. In the p.H4014T variant, it is assumed that the hydrogen bonds with C4032, C4035, and C4012 are lost due to the substitution of H4014 with T4014. In contrast, the p.R5153H variant shows no structural changes compared to the WT. AAA, ATPases associated with diverse cellular activities; RING, really interesting new gene; ZF2, NFX1 (nuclear transcription factor, X‐box binding 1)‐type zinc finger; CTD, C‐terminal domain.

The p.R4810K heterozygous or homozygous variants (1 patient) of *RNF213* were detected in 81 (26.6%) of the 305 MMD patients. Targeted resequencing of exons in the C‐terminal region of *RNF213* identified 14 RVs, including p.M3891I, p.R3940C, p.E3950G, p.H4014T, p.N4066S, p.M4289I, p.E4637K, p.G4640R, p.E4652V, p.D4863N, p.E4950D, p.S5012R, p.A5021V, and p.R5153H (Figure 4A). Among these RVs, seven are predicted to be benign, and seven are predicted to be probably damaging according to PolyPhen‐2 analysis (Table S4).

A 3D structural model of residues 520–5207 of human RNF213 was generated through homology modeling (Figure 4B). The p.R4810K variant and other C‐terminal RVs were mapped, with five variants located in the shell domain, three in the back domain, three in the C‐terminal domain, three in the core domain, and one in the ring domain. For each variant, mutant models were constructed to predict the impact of the mutations on the 3D structure (Figure 4C; Figure S5). Molecular modeling revealed that the p.R3940C, p.E3950G, and p.E4950D variants had missing salt bridges, while the p.M3891I, p.E4637K, p.R4810K, p.S5012R, and p.A5021V variants showed reduced salt bridges. The p.H4014T and p.G4640R variants exhibited missing hydrogen bonds, whereas the p.M4289I, p.G4640R, p.E4652V, p.D4863N, and p.R5153H variants showed no structural changes.

## 4 Discussion

In this study, the characteristics, predictors, and clinical significance of postoperative PA regression in a large cohort study of patients with MMD were analyzed. We found that revascularization surgery can promote PA regression in MMD, with distinct characteristics emerging across different PA subtypes. Additionally, five independent predictors associated with PA regression were identified. Importantly, the risk of hemorrhage was significantly higher in patients without PA regression than in those with PA regression, suggesting that PA regression may serve as a radiological marker for predicting long‐term hemorrhagic risk in MMD patients. Finally, through analysis of RNF213 homology modeling, we identified *RNF213* variants as being involved in the pathogenic mechanisms underlying PA evolution in MMD.

Previous reports demonstrated a reduction in abnormal net‐like vessels derived from the internal carotid artery and PCA following bypass surgery, particularly in pediatric patients [32, 33]. However, these studies assessed postoperative regression of moyamoya vessels as a whole, without differentiating them based on their anatomical aspects. Recent research suggests that abnormally dilated collateral vessels, referred to as PA vessels, should be classified into LSA, thalamic artery (THA), and choroidal artery (ChoA) and evaluated separately, as their pathophysiological significance varies [3, 4]. Therefore, in 2019, Miyakoshi et al. [7] were the first to demonstrate significant regression of PA vessels originating from the LSA, THA, and ChoA after DB. Kobayashi et al. [8] found that PA vessels originating from the LSA were more likely to regress after IB (*p* = 0.014), whereas those from the THA or ChoA showed little to no change. In contrast, a more recent pediatric series revealed a significant reduction in the choroidal PA score (*p* < 0.001) following IB, with no significant changes observed in the LSA (*p* = 0.243) and THA (*p* = 0.758) [9].

In our cohort, 103 (33.8%) of 305 hemispheres demonstrated PA regression after revascularization, while only 10 (3.3%) nonsurgical hemispheres exhibited regression. This suggests that revascularization promotes PA regression in the surgical hemisphere, with some, albeit limited, effects on the contralateral nonsurgical hemisphere. Notably, individual cases of PA progression were identified in both surgical and nonsurgical hemispheres, potentially attributed to the natural course of the disease or the effects of revascularization. On the other hand, focusing on the changes in each PA subtype after surgery, we found that the LSA, AChoA, and TTA were more likely to regress, while the TPA and PChoA were less prone to such changes. The trend was consistent in both pediatric and adult patients. When the AChoA and PChoA, as well as the TTA and TPA, are considered as single variables, the LSA exhibited the highest likelihood of regression (*p* < 0.001), followed by the ChoA (*p* < 0.001), and lastly the THA (*p* = 0.009) (Figure S6). These findings differ somewhat from previous studies, potentially due to variations in sample size, revascularization strategies, or age distribution within the study population. A prior report by Miyakoshi et al. [34] identified significant differences in the cortical distribution patterns among the three types of PA. The LSA anastomosis exhibited more anterior flow towards the cortex, particularly the superior and inferior frontal sulci and the interhemispheric fissure, compared to the thalamic or the choroidal anastomoses. The phenomenon may help explain the inconsistencies in the postoperative regression characteristics of PA subtypes observed in our study. In this context, Funaki et al. [35] suggest that a targeted bypass strategy or a larger fronto‐temporo‐parietal craniotomy, encompassing the cortical area posterior to the central sulcus, may be recommended.

Although the exact pathophysiological mechanisms of MMD remain unclear, dysfunction of the RNF213 protein, particularly in its role as an E3 ubiquitin ligase, is considered a critical factor [36]. Both the p.R4810K variant and other *RNF213* RVs, previously reported to be associated with MMD in White populations, are clustered in the C‐terminal region [18]. In 2022, we found that the p.R4810K and other C‐terminal RVs were significantly associated with developing various PA subtypes [22]. Torazawa et al. [19] recently also confirmed that the p.R4810K variant is significantly associated with LSA and THA anastomoses but not ChoA anastomosis. The genotype–phenotype relationship in MMD is further complicated by racial variations in the *RNF213* gene (Table S5). Herein, we focus on the characteristics of postoperative PA regression and its clinical significance. Multivariate analysis revealed that *RNF213* variants and preoperative PA scores were associated with PA regression. Zheng et al. [9] observed that patients with the *RNF213* p.R4810K variant were more likely to show postoperative PA regression, although it was not statistically significant (*p* = 0.069). This suggests that *RNF213* variants not only associated with PA progression but may also contribute to the onset of PA regression. Thus, the strong association between *RNF213* variants and preoperative PA scores with PA regression well reflects the dynamic evolution of PA before and after surgery. Additionally, to further explore the relationship between genotype and PA phenotype, a 3D model of human RNF213 was constructed. We found that mutations inducing conformational changes are more likely to lead to PA regression, suggesting that altered protein stability or disrupted interaction networks may influence vascular homeostasis.

Postoperative PA regression was also associated with age at operation, Suzuki stage, and Matsushima grade in this study. Our results indicate that younger patients are more susceptible to postoperative PA regression, and the trend appeared to decrease with age. This underscores the necessity of early revascularization for young patients with MMD, particularly those with significant PA dilation. However, Zheng et al. [9] observed that patients with PA progression were relatively younger than those with PA regression, although the difference was not statistically significant. The discrepancy may be attributed to the fact that their study focused exclusively on pediatric patients, while our study included both adult and pediatric patients. As expected, our study also demonstrated that PA is more prone to regress in Suzuki stages III–IV, likely due to the abundance of periventricular vessels during these stages, which gradually diminish in later stages. Yamamoto et al. [37] proposed a novel concept suggesting that, as the disease progresses and patients age, anastomosis vessels shift from the anterior to the posterior component. Interestingly, in our current cohort, postoperative PA regression also appears to follow an anterior‐to‐posterior pattern. The LSA and AChoA are the most likely to regress, followed by the posterior communicating artery (which may correspond to the thalamic PA), and finally the PChoA. The phenomenon indicates that PA regression may not only be the direct result of surgical intervention but also reflects the dynamic changes in the collateral network throughout the natural course of the disease.

**FIGURE 5:**
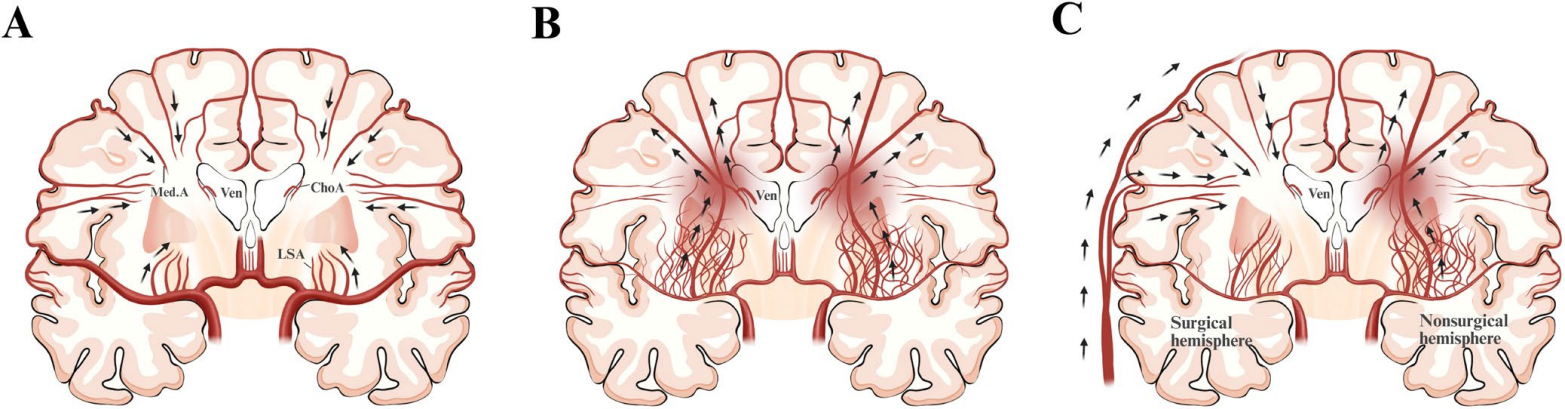
Schematic illustration of the coronal plane of the cerebral hemisphere and PA. (A) Normal cerebral hemispheres with normal blood flow. The black arrows indicate the direction of arterial blood flow. (B) Schematic illustration of lenticulostriate and choroidal anastomoses. Note that the direction of blood flow in the medullary artery shifts to centrifugal flow. The redness represents dilated and extended anastomotic vessels with bleeding. (C) Lenticulostriate and choroidal anastomoses regress in the left hemisphere, which underwent bypass surgery (the surgical hemisphere), while they remain present in the right hemisphere, which did not receive surgery (the nonsurgical hemisphere). Note that blood flow in the medullary artery of the surgical hemisphere has returned to normal. PA, periventricular anastomosis; LV, lateral ventricle; Med.A, medullary artery; and ChoA, choroidal artery.

**FIGURE 6:**
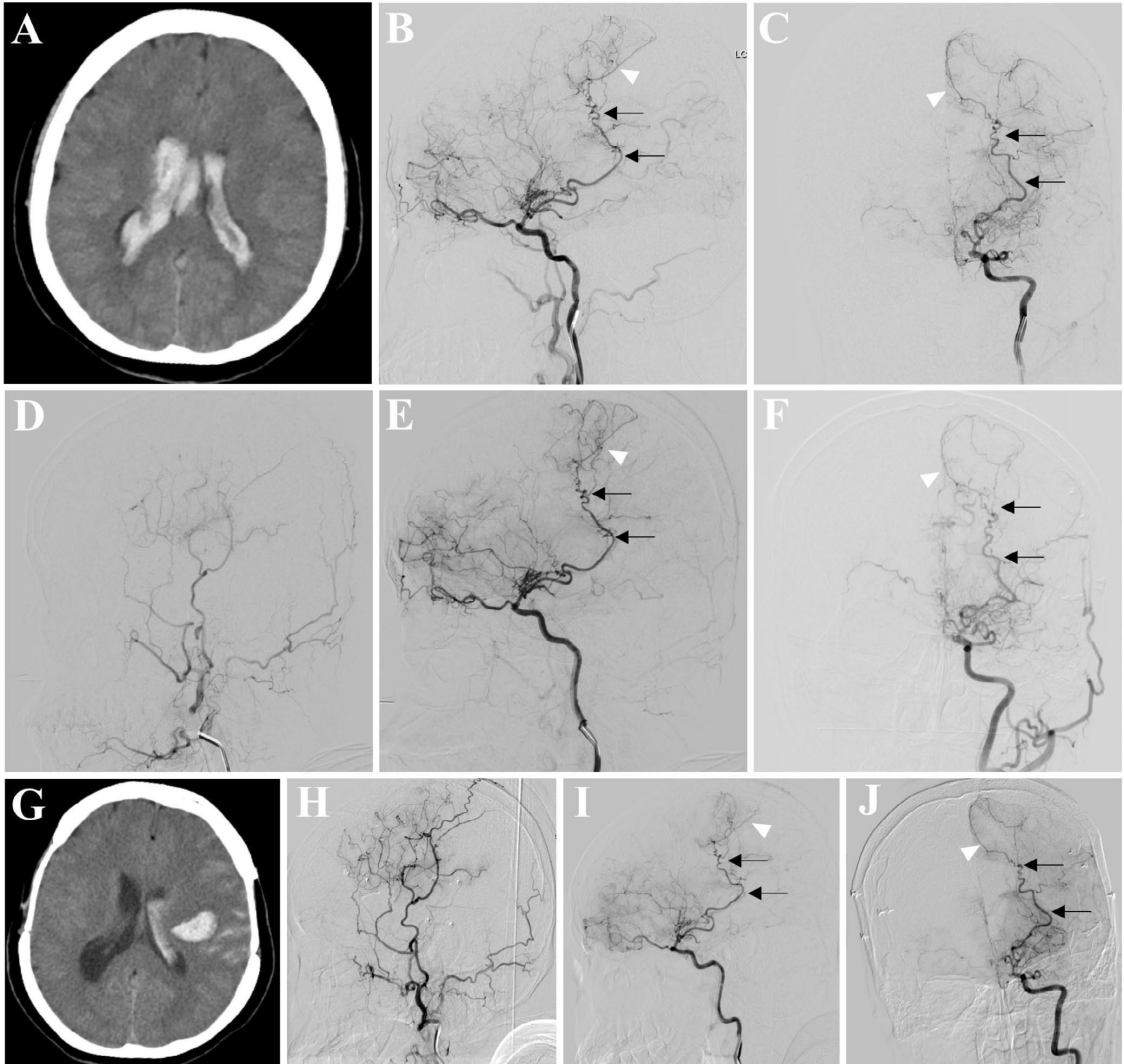
Representative case of rebleeding occurred during follow‐up without PA regression. (A) A 50‐year‐old woman with *RNF213* wild type suffered a sudden onset of intracranial ventricular hemorrhage. (B and C) Lateral (B) and anteroposterior (C) views of baseline left internal carotid angiography show typical findings of choroidal anastomosis, with extreme dilation and extension of the AChoA (black arrows) connecting to the medullary artery (white arrowheads). (D–F) Follow‐up angiography conducted 6 months after surgery showed a poor Matsushima grade (D) and no regression of the dilated and extension of the AChoA (E and F). (G–J) 4.7 years later, the patient suddenly experienced headache and weakness in the right limbs. CT scan revealed de novo hemorrhage located in the left periventricular area of the atrium of the lateral ventricle, consistent with the distribution of the choroidal anastomosis (G). Although subsequent angiography indicated an improvement in the Matsushima grade compared to the previous assessment (H), the dilated and extension of the AChoA had still not regression (I and J).

Consistent with findings from other studies, we noted that MMD patients with good PCF were more likely to exhibit PA regression [7, 8, 9, 32, 33]. The concepts of PA can reasonably explain this relationship (Figure 5). In MMD, the medial end of the medullary artery connects to the perforating arteries, and blood flow in the medullary artery is reversed to supply the cortex (Figure 5B). Once robust collateral flow is established through revascularization, the retrograde blood flow in the medullary artery can be restored to its normal direction, leading to PA regression and the normalization of perforating arteries (Figure 5C). Notably, the *RNF213* variants are associated with improved PCF, as demonstrated in Figure S7. We therefore propose that *RNF213* variants may promote favorable PCF, potentially contributing to the regression of pathological anastomoses. Additionally, a higher incidence of bleeding was observed in the non‐PA regression group compared to the PA regression group. This finding indicates that revascularization cannot always eliminate the abnormal collateral vessels, and these dilated, fragile PA vessels are likely an essential source of bleeding (Figure 6). However, given the relatively low hemorrhagic event rate in this cohort, the absence of hemorrhage in the PA regression group should be interpreted with caution, as this observation may partly be due to chance. Nevertheless, when evaluating surgical efficacy, attention should be given to both the degree of PCF and the regression of PA vessels.

## 5 Limitations and Strengths

Several limitations of our study should be noted. First, this is a nonrandomized cohort study with a relatively small sample size. Although selection bias was unavoidable, to our knowledge, this represents the largest sample size to date investigating PA regression in MMD. Second, the study evaluated two common revascularization modalities, CB and IB. However, DB is also performed in clinical practice, and some authors have reported its effects on collateral vessels [7, 38]. A multicenter prospective trial comparing various revascularization interventions is warranted. Lastly, the mean follow‐up time of 3.8 years in our study is relatively short and may not adequately capture long‐term stroke events. It is well established that recurrent hemorrhage can occur more than 10 years after the initial attack [39]. Therefore, patients enrolled in our study should be monitored for a longer duration to better determine the relationship between the postoperative PA regression and hemorrhage.

## 6 Conclusions

In conclusion, our results indicate that revascularization surgery can facilitate the PA regression in MMD, with distinct characteristics emerging across different PA subtypes. Furthermore, our study confirmed that *RNF213* variants in the C‐terminal region are associated with PA regression, underscoring a broader genotype–phenotype correlation. Importantly, PA regression could serve as a potential radiological marker for predicting the risk of future hemorrhage in MMD patients. These findings will contribute to elucidating the complex pathological mechanisms of MMD and may ultimately improve therapeutic decision‐making for this enigmatic disorder.

## Author Contributions

Conception and design: Jizong Zhao, Qian Zhang, Youyuan Bao. Acquisition of data: Qianjun Zhao, Xudong Sun, Fanbo Meng, Haoyuan Chen, Yunhao Cui, Qi Yuan. Analysis and interpretation of data: Youyuan Bao, Chaofan Zeng, Hao Li. Drafting the article: Youyuan Bao. Critically revising the article: Chaofan Zeng, Yimeng Xue, Dong Zhang, Rong Wang, Yan Zhang, Qian Zhang. Statistical analysis: Youyuan Bao, Chaofan Zeng. All authors have read and approved the final manuscript.

## Funding

This work was supported by the National Natural Science Foundation of China (82371295), and the National Key Research and Development Program of China (2021YFC2500502).

## Disclosure

The authors have nothing to report.

## Ethics Statement

The study was approved by the institutional review board and ethics committee of the Beijing Tiantan Hospital (approval number: KY2017‐141‐01 and KY2022‐051‐02), and written informed consent was obtained from all patients or their guardians.

## Conflicts of Interest

The authors declare no conflicts of interest.

## Supporting information


**Figure S1:** Evaluation of postoperative collateral formation by using the Matsushima scale.
**Figure S2:** Representative angiograms of each PA subtype and the corresponding postoperative PA changes.
**Figure S3:** PA score comparison between the surgical and nonsurgical hemispheres before and after revascularization surgery.
**Figure S4:** Comparison of angiographic changes in PA subtypes before and after revascularization surgery in both pediatric and adult patients.
**Figure S5:** Structural changes of other C‐terminal rare variants in our series.
**Figure S6:** Angiographic changes in different PA subtypes before and after revascularization surgery.
**Figure S7:** Comparison of postoperative Matsushima grading across groups with different *RNF213* variants.
**Table S1:** Distribution of postoperative PA changes in all 610 hemispheres.
**Table S2:** Interrater agreement for periventricular anastomosis subtype.
**Table S3:** Results of multicollinearity analysis.
**Table S4:** Characteristics of each *RNF213* variant identified in this study.
**Table S5:** Frequency of *RNF213* p.R4810K variant and RVs in patients with moyamoya disease in different countries.

## Data Availability

The data that support the findings of this study are available from the corresponding author upon reasonable request.

## References

[1] R. M. Scott and E. R. Smith , “Moyamoya Disease and Moyamoya Syndrome,” New England Journal of Medicine 360 (2009): 1226–1237. 19297575 10.1056/NEJMra0804622

[2] S. Kuroda and K. Houkin , “Moyamoya Disease: Current Concepts and Future Perspectives,” Lancet Neurology 7 (2008): 1056–1066. 18940695 10.1016/S1474-4422(08)70240-0

[3] T. Funaki , Y. Fushimi , J. C. Takahashi , et al., “Visualization of Periventricular Collaterals in Moyamoya Disease With Flow‐Sensitive Black‐Blood Magnetic Resonance Angiography: Preliminary Experience,” Neurologia Medico‐Chirurgica (Tokyo) 55 (2015): 204–209. 10.2176/nmc.oa.2014-0360 PMC4533333 25739429

[4] T. Funaki , J. C. Takahashi , K. Houkin , et al., “Angiographic Features of Hemorrhagic Moyamoya Disease With High Recurrence Risk: A Supplementary Analysis of the Japan Adult Moyamoya Trial,” Journal of Neurosurgery 128 (2018): 777–784. 28409736 10.3171/2016.11.JNS161650

[5] T. Funaki , J. C. Takahashi , K. Houkin , et al., “Effect of Choroidal Collateral Vessels on de Novo Hemorrhage in Moyamoya Disease: Analysis of Nonhemorrhagic Hemispheres in the Japan Adult Moyamoya Trial,” Journal of Neurosurgery 132 (2020): 408–414. 30738387 10.3171/2018.10.JNS181139

[6] T. Funaki , J. C. Takahashi , K. Houkin , et al., “High Rebleeding Risk Associated With Choroidal Collateral Vessels in Hemorrhagic Moyamoya Disease: Analysis of a Nonsurgical Cohort in the Japan Adult Moyamoya Trial,” Journal of Neurosurgery 130 (2019): 525–530. 29498573 10.3171/2017.9.JNS17576

[7] A. Miyakoshi , T. Funaki , J. C. Takahashi , et al., “Restoration of Periventricular Vasculature After Direct Bypass for Moyamoya Disease: Intra‐Individual Comparison,” Acta Neurochirurgica 161 (2019): 947–954. 30880348 10.1007/s00701-019-03866-9

[8] M. Kobayashi , Y. Akamatsu , K. Chida , et al., “Changes in Periventricular Anastomosis After Indirect Revascularization Surgery Alone for Adult Patients With Misery Perfusion due to Ischemic Moyamoya Disease,” Neurosurgical Review 45 (2022): 3665–3673. 36112252 10.1007/s10143-022-01861-w

[9] E. Y. Zheng , S. Hara , M. Inaji , Y. Tanaka , T. Nariai , and T. Maehara , “Regression of Periventricular Anastomosis After Indirect Revascularization in Pediatric Patients With Moyamoya Disease,” Journal of Neurosurgery. Pediatrics 32 (2023): 719–728. 37773770 10.3171/2023.8.PEDS23304

[10] S. Yamamoto , D. Kashiwazaki , H. Uchino , et al., “Ameliorative Effects of Combined Revascularization Surgery on Abnormal Collateral Channels in Moyamoya Disease,” Journal of Stroke and Cerebrovascular Diseases 30 (2021): 105624. 33516067 10.1016/j.jstrokecerebrovasdis.2021.105624

[11] H. Yamada , T. Funaki , Y. Fushimi , et al., “Early Radiological Reduction of Periventricular Anastomosis After Direct Bypass Surgery for Adult Moyamoya Disease,” Journal of Neurosurgery 1‐9 (2024): 1428–1436. 10.3171/2024.7.JNS24237 39729585

[12] S. Miyamoto , T. Yoshimoto , N. Hashimoto , et al., “Effects of Extracranial‐Intracranial Bypass for Patients With Hemorrhagic Moyamoya Disease: Results of the Japan Adult Moyamoya Trial,” Stroke 45 (2014): 1415–1421. 24668203 10.1161/STROKEAHA.113.004386

[13] W. Liu , D. Morito , S. Takashima , et al., “Identification of RNF213 as a Susceptibility Gene for Moyamoya Disease and Its Possible Role in Vascular Development,” PLoS One 6 (2011): e22542. 21799892 10.1371/journal.pone.0022542 PMC3140517

[14] F. Kamada , Y. Aoki , A. Narisawa , et al., “A Genome‐Wide Association Study Identifies RNF213 as the First Moyamoya Disease Gene,” Journal of Human Genetics 56 (2011): 34–40. 21048783 10.1038/jhg.2010.132

[15] Q. Zhang , Y. Liu , D. Zhang , et al., “RNF213 as the Major Susceptibility Gene for Chinese Patients With Moyamoya Disease and Its Clinical Relevance,” Journal of Neurosurgery 126 (2017): 1106–1113. 27128593 10.3171/2016.2.JNS152173

[16] T. Ok , Y. H. Jung , and K. Y. Lee , “Genotype‐Phenotype Correlation of the *RNF213* r4810k Variant in Moyamoya Disease,” Journal of Stroke 25 (2023): 303–306. 36916017 10.5853/jos.2023.00297 PMC10250872

[17] P. Ge , X. Ye , X. Liu , et al., “Association Between p.r4810k Variant and Postoperative Collateral Formation in Patients With Moyamoya Disease,” Cerebrovascular Diseases 48 (2019): 77–84. 31578010 10.1159/000503250

[18] S. Guey , M. Kraemer , D. Herve , et al., “Rare RNF213 Variants in the c‐Terminal Region Encompassing the RING‐Finger Domain Are Associated With Moyamoya Angiopathy in Caucasians,” European Journal of Human Genetics 25 (2017): 995–1003. 28635953 10.1038/ejhg.2017.92 PMC5567158

[19] S. Torazawa , S. Miyawaki , H. Imai , et al., “RNF213 p.arg4810lys Wild Type Is Associated With de Novo Hemorrhage in Asymptomatic Hemispheres With Moyamoya Disease,” Translational Stroke Research 15 (2024): 729–738. 37269436 10.1007/s12975-023-01159-z PMC11226534

[20] A. C. Cecchi , D. Guo , Z. Ren , et al., “RNF213 Rare Variants in an Ethnically Diverse Population With Moyamoya Disease,” Stroke 45 (2014): 3200–3207. 25278557 10.1161/STROKEAHA.114.006244 PMC4420622

[21] S. Nomura , H. Akagawa , K. Yamaguchi , et al., “Difference in Clinical Phenotype, Mutation Position, and Structural Change of RNF213 Rare Variants Between Pediatric and Adult Japanese Patients With Moyamoya Disease,” Translational Stroke Research 15 (2024): 1142–1153. 37768541 10.1007/s12975-023-01194-w

[22] Y. Xue , C. Zeng , P. Ge , et al., “Association of RNF213 Variants With Periventricular Anastomosis in Moyamoya Disease,” Stroke 53 (2022): 2906–2916. 35543128 10.1161/STROKEAHA.121.038066

[23] Guidelines for Diagnosis and Treatment of Moyamoya Disease (Spontaneous Occlusion of the Circle of Willis),” Neurologia Medico‐Chirurgica (Tokyo) 52 (2012): 245–266. 10.2176/nmc.52.245 22870528

[24] Y. Ma , M. Li , L. Q. Jiao , H. Q. Zhang , and F. Ling , “Contralateral Cerebral Hemodynamic Changes After Unilateral Direct Revascularization in Patients With Moyamoya Disease,” Neurosurgical Review 34, no. 347–353 (2011): 353–354. 10.1007/s10143-011-0312-y 21538064

[25] X. Deng , F. Gao , D. Zhang , et al., “Effects of Different Surgical Modalities on the Clinical Outcome of Patients With Moyamoya Disease: A Prospective Cohort Study,” Journal of Neurosurgery 128 (2018): 1327–1337. 28686113 10.3171/2016.12.JNS162626

[26] M. Kircher , D. M. Witten , P. Jain , B. J. O'Roak , G. M. Cooper , and J. Shendure , “A General Framework for Estimating the Relative Pathogenicity of Human Genetic Variants,” Nature Genetics 46 (2014): 310–315. 24487276 10.1038/ng.2892 PMC3992975

[27] M. Morioka , J. Hamada , T. Kawano , et al., “Angiographic Dilatation and Branch Extension of the Anterior Choroidal and Posterior Communicating Arteries Are Predictors of Hemorrhage in Adult Moyamoya Patients,” Stroke 34 (2003): 90–95. 12511756 10.1161/01.str.0000047120.67507.0d

[28] Z. W. Liu , C. Han , F. Zhao , et al., “Collateral Circulation in Moyamoya Disease: A New Grading System,” Stroke 50 (2019): 2708–2715. 31409266 10.1161/STROKEAHA.119.024487

[29] H. Yin , X. Liu , D. Zhang , et al., “A Novel Staging System to Evaluate Cerebral Hypoperfusion in Patients With Moyamoya Disease,” Stroke 49 (2018): 2837–2843. 30571396 10.1161/STROKEAHA.118.022628

[30] T. Matsushima , T. Inoue , S. O. Suzuki , K. Fujii , M. Fukui , and K. Hasuo , “Surgical Treatment of Moyamoya Disease in Pediatric Patients–Comparison Between the Results of Indirect and Direct Revascularization Procedures,” Neurosurgery 31 (1992): 401–405. 1407421 10.1227/00006123-199209000-00003

[31] J. Ahel , A. Lehner , A. Vogel , et al., “Moyamoya Disease Factor RNF213 Is a Giant e3 Ligase With a Dynein‐Like Core and a Distinct Ubiquitin‐Transfer Mechanism,” eLife 9 (2020): 9. 10.7554/eLife.56185 PMC7311170 32573437

[32] I. Yamada , Y. Matsushima , and S. Suzuki , “Childhood Moyamoya Disease Before and After Encephalo‐Duro‐Arterio‐Synangiosis: An Angiographic Study,” Neuroradiology 34 (1992): 318–322. 1528443 10.1007/BF00588191

[33] K. Irikura , Y. Miyasaka , A. Kurata , et al., “The Effect of Encephalo‐Myo‐Synangiosis on Abnormal Collateral Vessels in Childhood Moyamoya Disease,” Neurological Research 22 (2000): 341–346. 10874680 10.1080/01616412.2000.11740680

[34] A. Miyakoshi , T. Funaki , Y. Fushimi , et al., “Cortical Distribution of Fragile Periventricular Anastomotic Collateral Vessels in Moyamoya Disease: An Exploratory Cross‐Sectional Study of Japanese Patients With Moyamoya Disease,” AJNR. American Journal of Neuroradiology 41 (2020): 2243–2249. 33154076 10.3174/ajnr.A6861 PMC7963258

[35] T. Funaki , A. Miyakoshi , H. Kataoka , et al., “Larger Posterior Revascularization Associated With Reduction of Choroidal Anastomosis in Moyamoya Disease: A Quantitative Angiographic Analysis,” AJNR. American Journal of Neuroradiology 43 (2022): 1279–1285. 36007950 10.3174/ajnr.A7609 PMC9451642

[36] F. Ye , X. Niu , F. Liang , et al., “RNF213 Loss‐Of‐Function Promotes Pathological Angiogenesis in Moyamoya Disease via the Hippo Pathway,” Brain 146 (2023): 4674–4689. 37399508 10.1093/brain/awad225 PMC10629795

[37] S. Yamamoto , S. Hori , D. Kashiwazaki , N. Akioka , N. Kuwayama , and S. Kuroda , “Longitudinal Anterior‐To‐Posterior Shift of Collateral Channels in Patients With Moyamoya Disease: An Implication for Its Hemorrhagic Onset,” Journal of Neurosurgery 130 (2019): 884–890. 29570010 10.3171/2017.9.JNS172231

[38] H. Jiang , W. Ni , B. Xu , et al., “Outcome in Adult Patients With Hemorrhagic Moyamoya Disease After Combined Extracranial‐Intracranial Bypass,” Journal of Neurosurgery 121 (2014): 1048–1055. 25127415 10.3171/2014.7.JNS132434

[39] Q. Zhang , Z. Yin , C. Zhu , et al., “Effectiveness of Indirect Revascularization for Adult Hemorrhagic Moyamoya Disease: A 10‐Year Follow‐Up Study,” Journal of Neurosurgery 140 (2024): 764–773. 37877987 10.3171/2023.6.JNS23727

