# Supplementary material for: RNF213 Variants Associated With Periventricular Anastomosis Regression After Revascularization in Moyamoya Disease
Source: CNS Neurosci Ther. 2026 Jun 12;32(6):e70982. doi: 10.1002/cns.70982 (PMC13263247; doi:10.1002/cns.70982)
Supplement: Supplementary file 1 — Figure S1: Evaluation of postoperative collateral formation by using the Matsushima scale. Figure S2: Representative angiograms of each PA subtype and the corresponding postoperative PA changes. Figure S3: PA score comparison between the surgical and nonsurgical hemispheres before and after revascularization surgery. Figure S4: Comparison of angiographic changes in PA subtypes before and after revascularization surgery in both pediatric and adult patients. Figure S5: Structural changes of other C‐terminal rare variants in our series. Figure S6: Angiographic changes in different PA subtypes before and after revascularization surgery. Figure S7: Comparison of postoperative Matsushima grading across groups with different RNF213 variants. Table S1: Distribution of postoperative PA changes in all 610 hemispheres. Table S2: Interrater agreement for periventricular anastomosis subtype. Table S3: Results of multicollinearity analysis. Table S4: Characteristics of each RNF213 variant identified in this study. Table S5: Frequency of RNF213 p.R4810K variant and RVs in patients with moyamoya disease in different countries. [file CNS-32-e70982-s001.docx]

**Supplementary Tables**

**Supplementary Table 1.** **Distribution of postoperative PA changes in all 610 hemispheres**

| Hemisphere | No. of  Hemispheres | PA | | |
| --- | --- | --- | --- | --- |
|  |  | Regression* | Unchanged | Progression |
| Surgical | 305 | 103 (33.8%) | 199 (65.2%) | 3 (1.0%) |
| Nonsurgical | 305 | 10 (3.3%) | 283 (92.8%) | 12 (3.9%) |

PA, periventricular anastomosis.

* *p*<0.001, chi-square test.

**Supplementary Table 2. Interrater agreement for periventricular anastomosis subtype**

| **Variable** | **Statistical methods** | **Pre** | | **Post** | |
| --- | --- | --- | --- | --- | --- |
|  |  | **Value** | **95% CI** | **Value** | **95% CI** |
| PA score | ICC | 0.83 | 0.78-0.87 | 0.82 | 0.77-0.86 |
| LSA grade | weighted kappa | 0.82 | 0.77-0.87 | 0.85 | 0.80-0.89 |
| TTA grade | weighted kappa | 0.75 | 0.68-0.81 | 0.76 | 0.69-0.82 |
| TPA grade | weighted kappa | 0.72 | 0.64-0.79 | 0.70 | 0.62-0.78 |
| AchoA grade | weighted kappa | 0.80 | 0.74-0.85 | 0.83 | 0.78-0.88 |
| PchoA grade | weighted kappa | 0.74 | 0.67-0.80 | 0.73 | 0.66-0.80 |

LSA, lenticulostriate artery; AChoA, anterior choroidal artery; PChoA, posterior choroidal artery; TPA, thalamoperforating artery; TTA, thalamotuberal artery; ICC, intraclass correlation coefficient

**Supplementary Table 3. Results of multicollinearity analysis**

| **Variables** | **VIF** |
| --- | --- |
| Age | 1.433 |
| Sex | 1.089 |
| Clinical manifestation | 1.138 |
| Suzuki stage | 1.472 |
| Collateral circulation | 1.544 |
| PCA involvement | 1.467 |
| Stage of the preinfarction period | 1.076 |
| Pre PA score | 1.514 |
| ECA collateral | 1.174 |
| Surgery type | 1.432 |
| Genotype of *RNF213* | 1.205 |
| Post Matsushima grading | 1.202 |

VIF, variance inflation factor; PCA, posterior cerebral artery; ECA, external carotid artery.

| **Position**  **(GRCh37)** | **rsID** | **Amino Acid**  **Change** | **gnomAD**  **WES Freq** | **Polyphen2**  **HumDiv** | **CADD**  **(GRCh37-v1.6)** | **REVEL**  **Score** |
| --- | --- | --- | --- | --- | --- | --- |
| 17:78337512  17:78338300  17:78338331  17:78341828  17:78343343  17:78346890  17:78355458  17:78355467  17:78355504  17:78358945  17:78360097  17:78360619  17:78363006  17:78363034  17:78363984 | rs1463105412  rs377419916  NA  NA  NA  rs374262786  NA  rs138223459  NA  rs112735431  rs760732823  rs371441113  rs771183046  rs138130613  rs528073196 | p.M3891I  p.R3940C  p.E3950G  p.H4014T  p.N4066S  p.M4289I  p.E4637K  p.G4640R  p.E4652V  p.R4810K  p.D4863N  p.E4950D  p.S5012R  p.A5021V  p.R5153H | 1.24*10^-6^  2.4*10^-5^  NA  NA  NA  3.2*10^-5^  NA  NA  NA  2.4*10^-4^  6.0*10^-5^  2.3*10^-4^  4.0*10^-6^  3.1*10^-4^  2.5*10^-5^ | Benign  PD  Benign  PD  PD  Benign  PD  Benign  Benign  PD  PD  PD  Benign  Benign  PD | 2.1  13.7  13.6  25.3  23.8  14.4  24.3  13.2  8.6  13.2  22.1  18.4  9.3  14.5  15.3 | 0.053  0.036  0.033  0.826  0.111  0.078  0.321  0.031  0.032  0.071  0.112  0.124  0.058  0.054  0.048 |

**Supplementary Table 4. Characteristics of each *RNF213* variant identified in this study**

gnom AD WES Freq = Gnome Aggregation Database Whole Exome Sequence Frequency; HumDiv = data set used by PolyPhen-2; CADD = combined annotated-dependent depletion; REVEL = Rare Exome Variant Ensemble Learner. PD = Probably Damaging; and NA = not applicable.

| **Authors & Year** | **Population** | **No. of Cases** | ***RNF213* p.R4810K** | | | ***RNF213* RVs** | |
| --- | --- | --- | --- | --- | --- | --- | --- |
|  |  |  | **n** | **Frequency (%)** | | **n** | **Frequency (%)** |
| Liu et al.,^1^ 2011  Wu et al.,^2^ 2012  Cecchi et al.,^3^ 2014  Moteki et al.,^4^ 2015 | Japanese  Korean  Chinese  Czech  German  Chinese  Mainly European  Japanese | 161  38  52  8  42  170  110  103 | 145  30  12  0  0  22  9*  76 | | 90.1  78.9  23.1  0.0  0.0  12.9  8.2  73.8 | 0  0  7  2  3  5  11  5 | 0.0  0.0  13.5  25.0  4.9  25.0  10.0  4.9 |
| Guey et al.,^5^ 2017 | European | 68 | 0 | | 0.0 | 5 | 7.4 |
| Jang et al et al.,^6^ 2017  Kobayashi et al.,^7^ 2017  Zhang et al.,^8^ 2017  Hara et al.,^9^ 2022^†^  Nomura et al.,^10^ 2023  Torazawa et al.,^11^ 2023  Current Study | Korean  European  Chinese  Japanese  Japanese  Japanese  Chinese | 264  18  255  129  151  139  305 | 178  0  80  104 ^‡^  121^§^  100  81** | | 67.4  0.0  31.4  80.6  80.1  71.9  26.6 | 2  4  27  8  16 ^\|\|^  15^#^  15 | 0.8  22.2  10.6  6.2  10.6  0.8  4.9 |

**Supplementary Table 5. Frequency of *RNF213* p.R4810K variant and RVs in patients with moyamoya disease in different countries**

RVs, rare variants.

^*^All 9 patients were of Asian descent.

^†^ All patients were children.

^‡^ There are 12 patients with *RNF213* p.R4810K homozygous.

^§^ There are 10 patients with *RNF213* p.R4810K homozygous.

^||^ 11 patients coexisted with *RNF213* p.R4810K heterozygotes.

^#^ 5 patients coexisted with *RNF213* p.R4810K heterozygotes.

^**^ There are 1 patient with *RNF213* p.R4810K homozygous.

**Supplementary Figures**

**
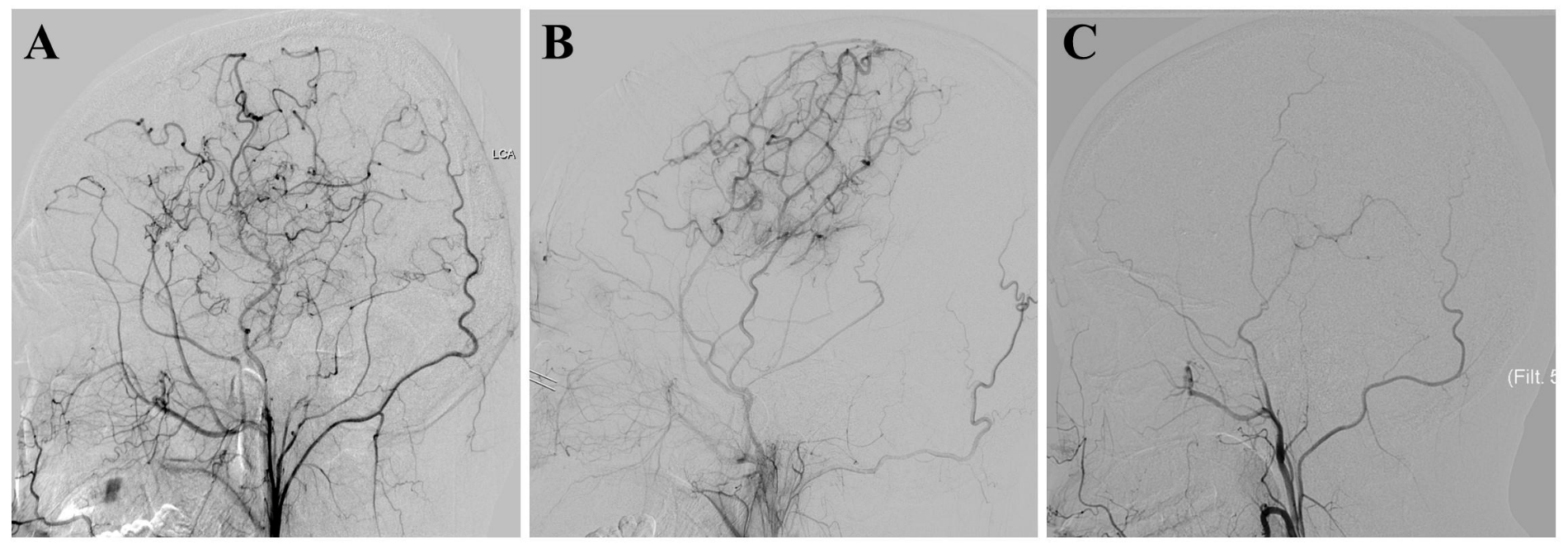
 Supplementary Figure 1.** Evaluation of postoperative collateral formation by using the Matsushima scale. **(A)** Grade A: collateral formation covering more than two-thirds of the middle cerebral artery (MCA) distribution. **(B)** Grade B: collateral formation covering between two-thirds and one-third of the MCA distribution. **(C)** Grade C: no obvious collateral formation.

**
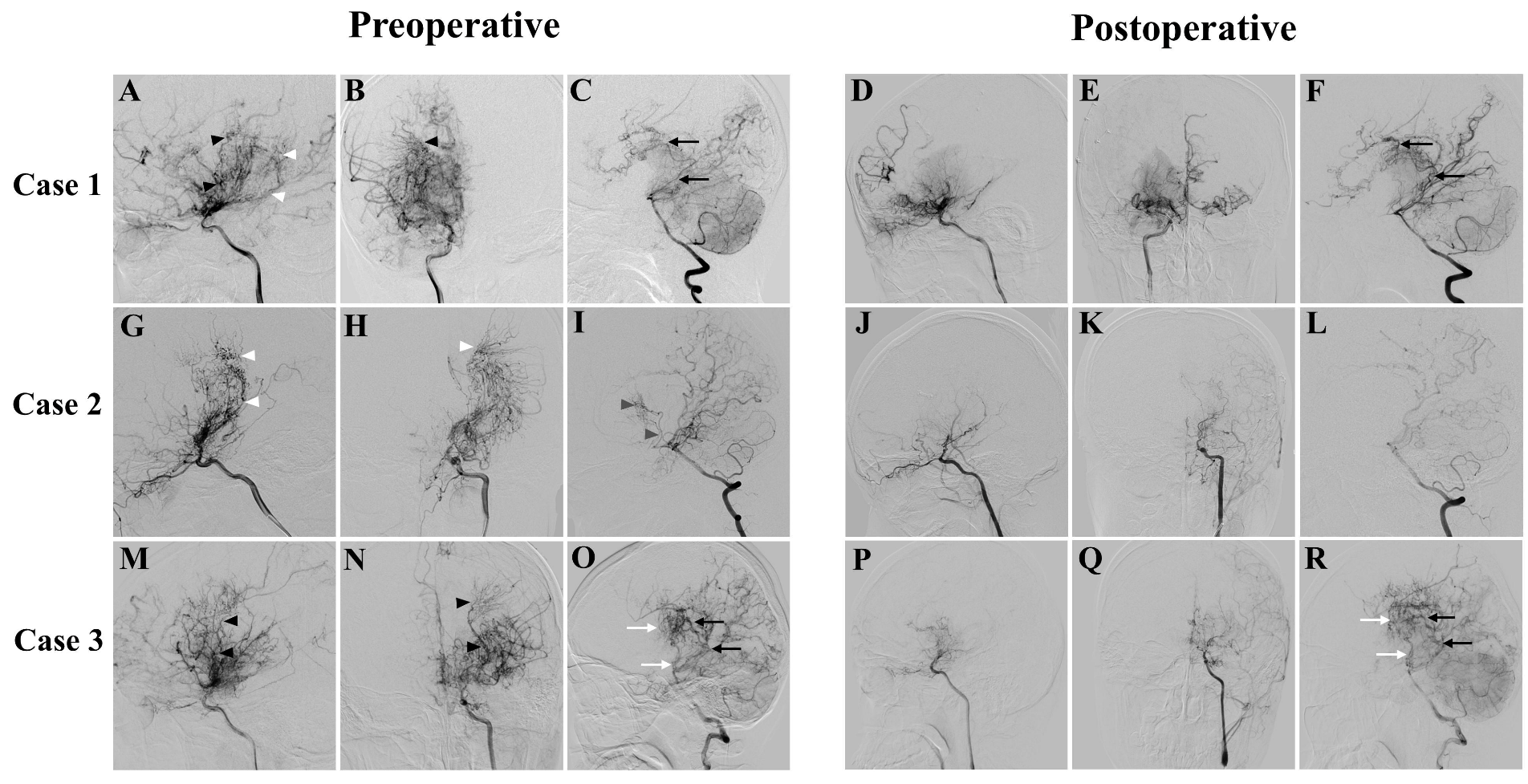
**

**Supplementary Figure 2.** Representative angiograms of each periventricular anastomosis (PA) subtype and the corresponding postoperative PA changes. **(A-I)** The left panel represented the preoperative angiograms, and the right panel **(J-R)** represented the corresponding postoperative angiograms. Note that the TPA and PChoA did not regress after surgery. The black arrows indicate dilated and extended LSA; The white arrows indicate dilated and extended AChoA; The gray arrows indicate dilated and extended TTA; The white arrowheads indicate dilated and extended TPA; The black arrowheads indicate dilated and extended PChoA.


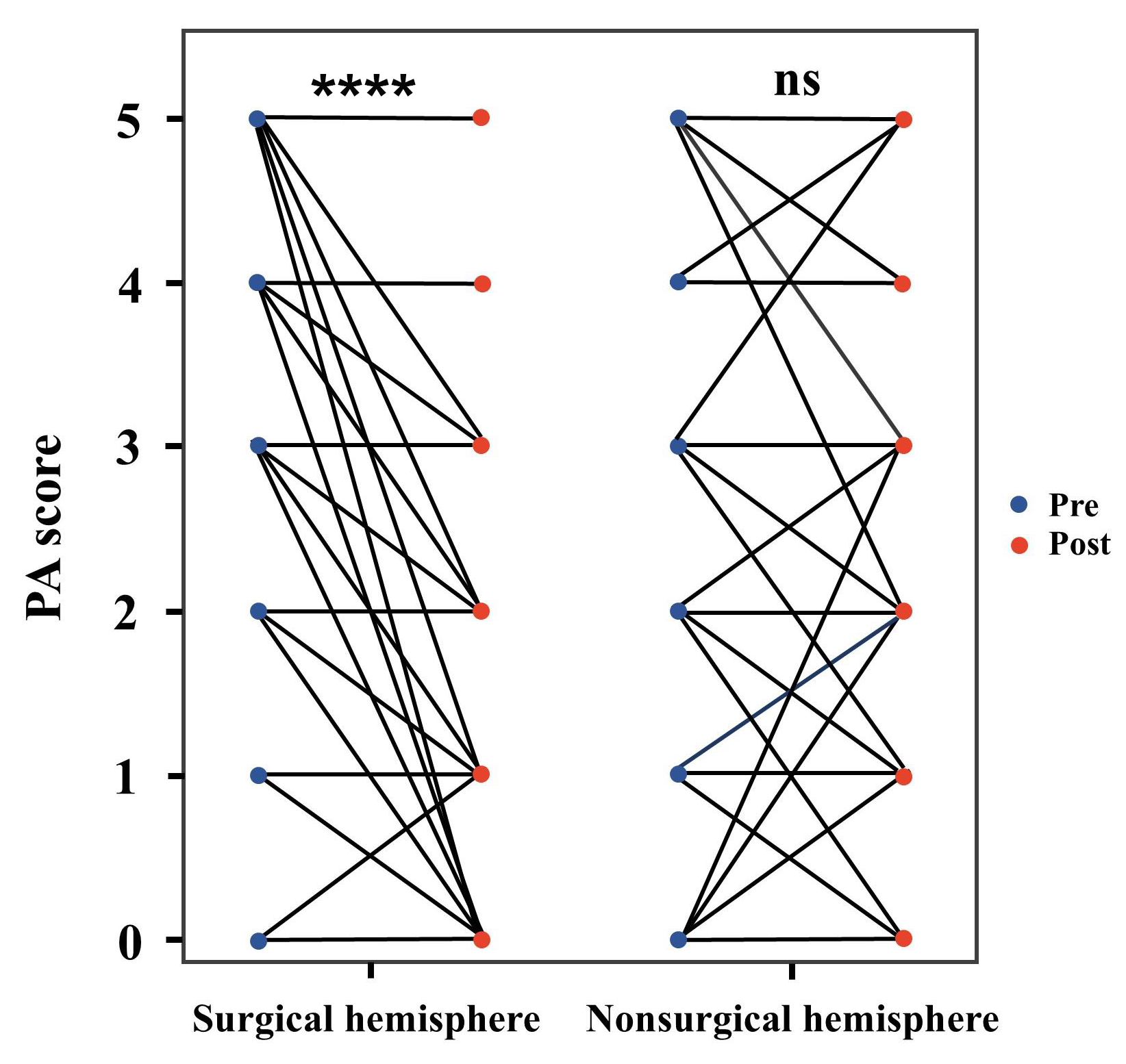


**Supplementary Figure 3.** PA score comparison between the surgical and nonsurgical hemispheres before and after revascularization surgery. Line graphs illustrate within-pair change in pre- and postoperative PA score in both the surgical and nonsurgical hemispheres (n = 610). The PA score significantly decreased in the surgical hemispheres compared to the nonsurgical hemispheres. **** *p*<0.0001 (Wilcoxon signed-rank test). Figure is available in color online only.

**
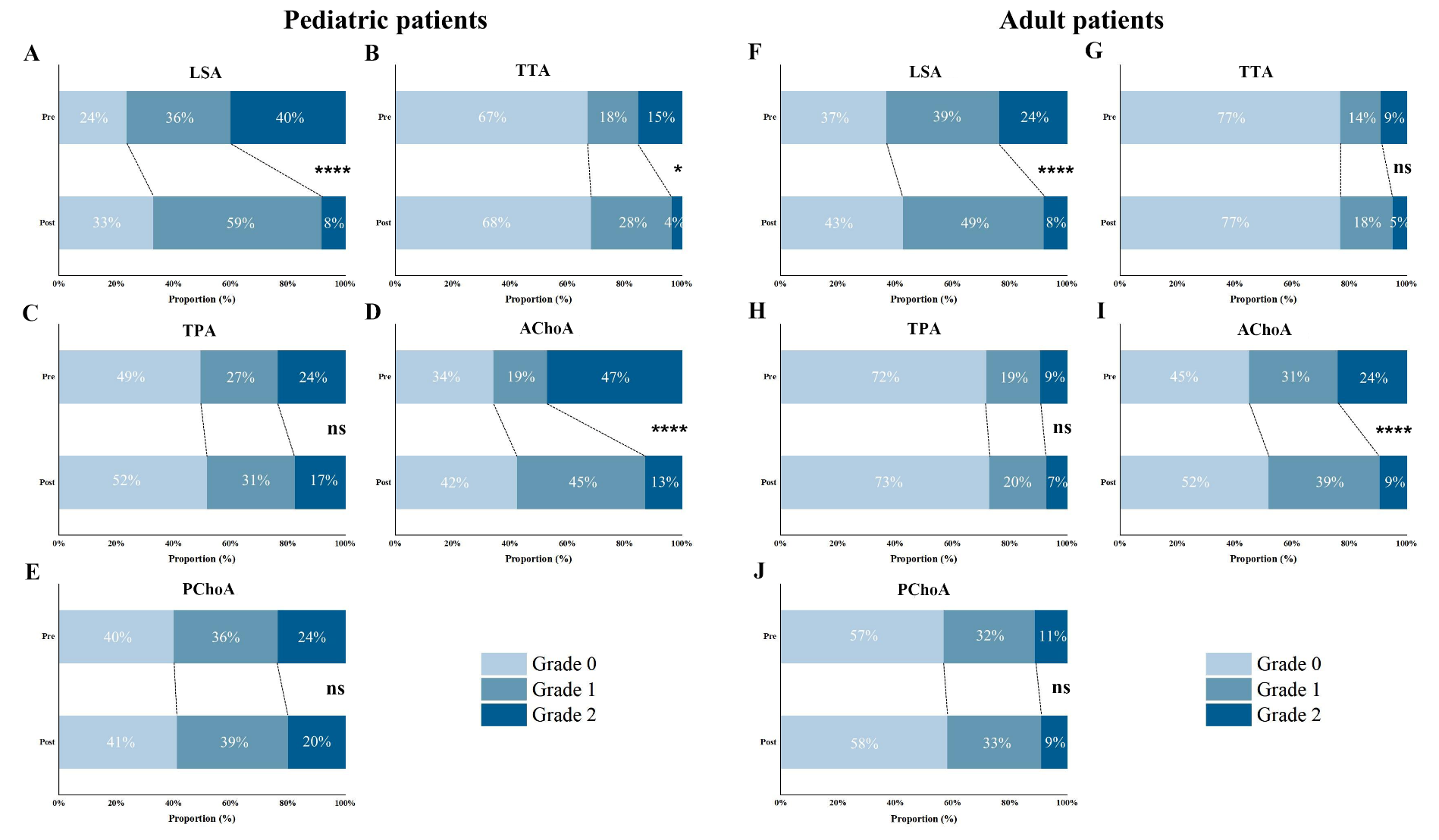
**

**Supplementary Figure 4.** Comparison of angiographic changes in PA subtypes before and after revascularization surgery in both pediatric and adult patients. In the pediatric group, LSA **(A)**, TTA **(B)**, and AChoA **(D)** exhibited significant regression, while TPA **(C)** and PChoA **(E)** showed no significant changes. In the adult group, LSA **(F)** and AChoA **(I)** exhibited significant regression, whereas TTA **(G)**, TPA **(H)**, and PChoA **(J)** showed no significant changes. **p*< 0.05; ***p*< 0.01; ****p*< 0.001; *****p*< 0.0001. Figure is available in color online only.

**
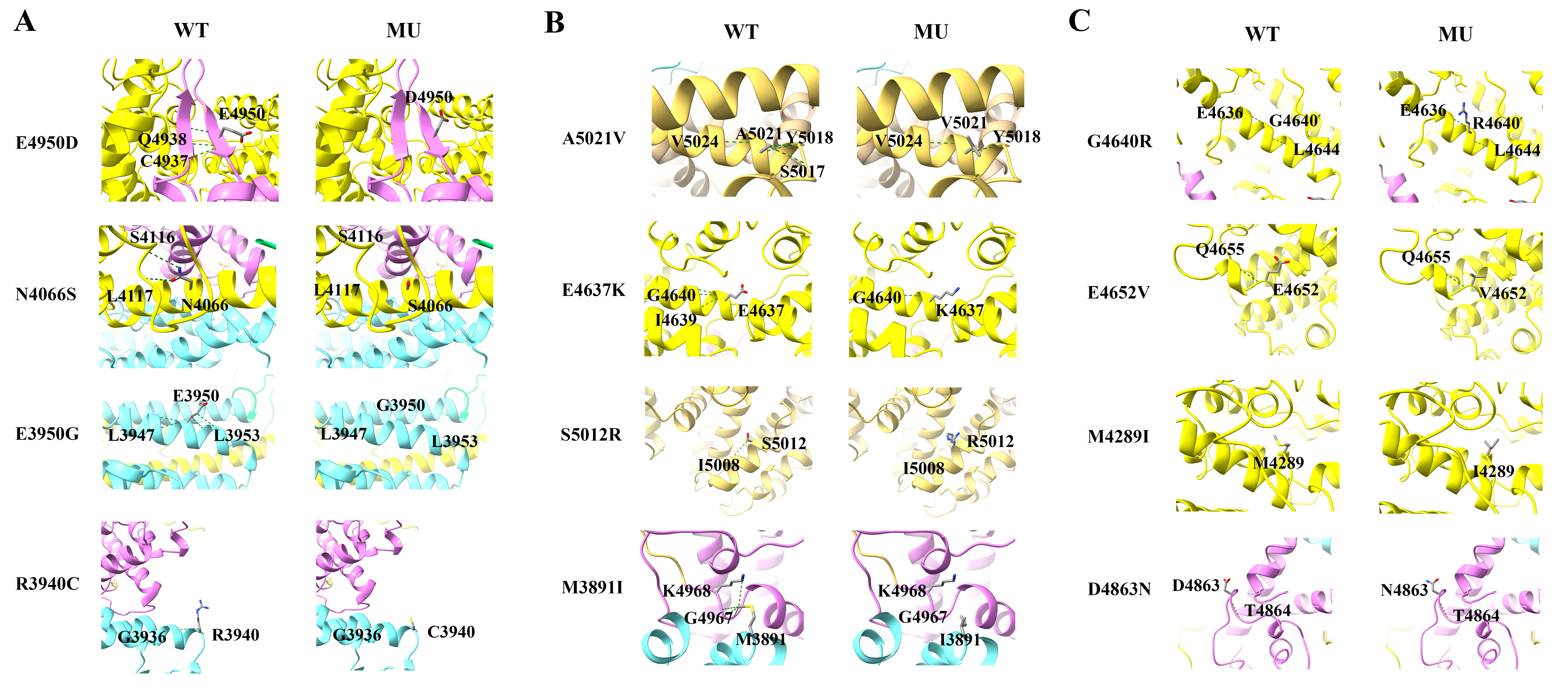
**

**Supplementary Figure 5.** Structural changes of other C-terminal rare variants in our series. **(A)** The p.N4066S variant exhibits a loss of hydrogen bonds, while the p.E4950D, p.E3950G, and p.R3940C variants exhibit a loss of salt bridges compared to the wild type (WT). **(B)** The p.A5021V, p.E4637K, p.S5012R, and p.M3891I variants show a reduction in salt bridges compared to WT. **(C)** The p.G4640R, p.E4652V, p.M4289I, and p.D4863N variants do not exhibit any structural changes compared to WT. MU, mutation. Figure is available in color online only.

**
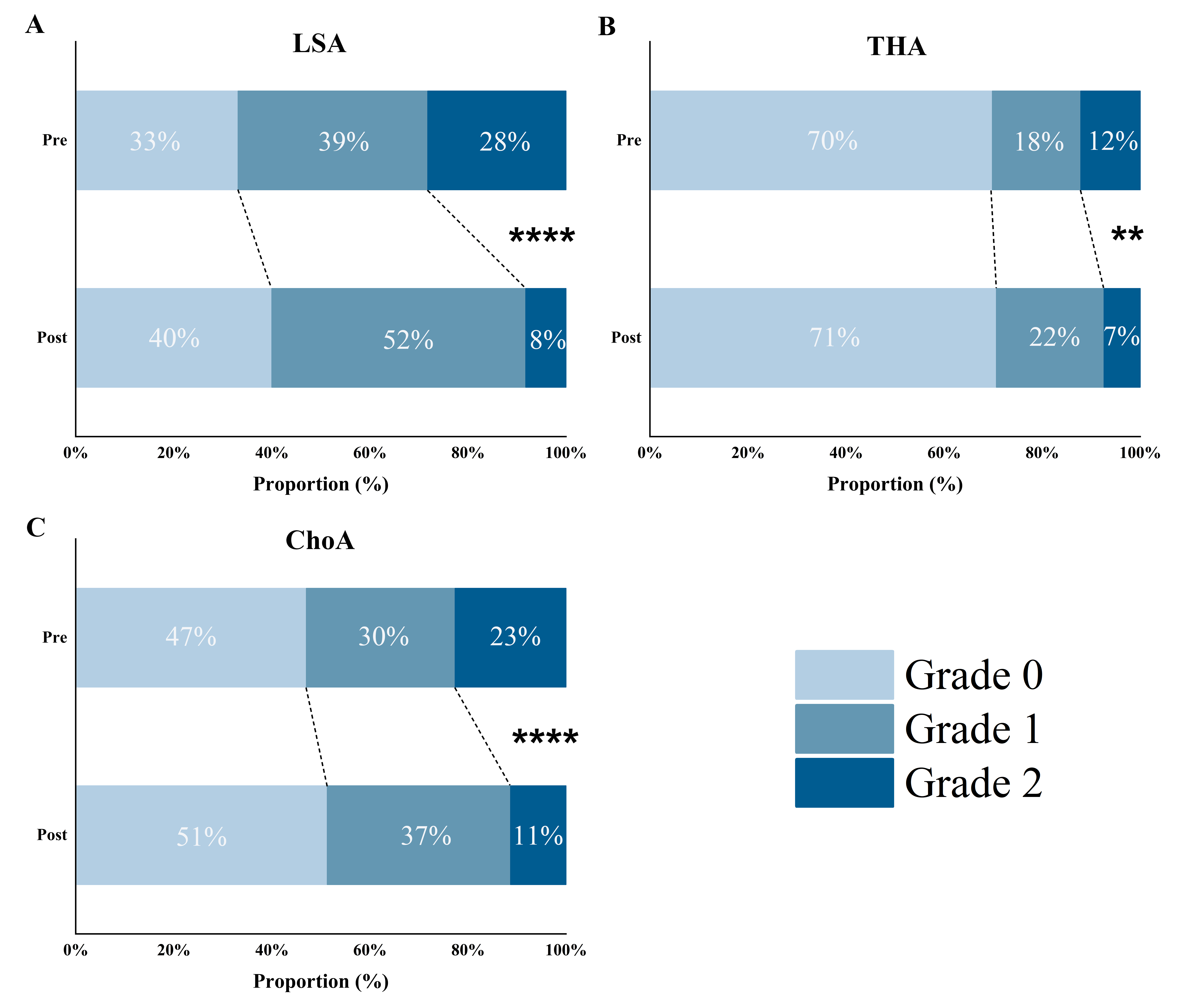
**

**Supplementary Figure 6.** Angiographic changes in different PA subtypes before and after revascularization surgery.

The stacked chart showed that the LSA **(A)**, THA **(B)**, and ChoA **(C)** showed significant regression (p<0.0001, *p*=0.009, and *p*<0.0001, respectively). ChoA, choroidal artery; THA, thalamic artery. **p*<0.05; ***p*<0.01; ****p*<0.001; **** *p*<0.0001. Figure is available in color online only.

**
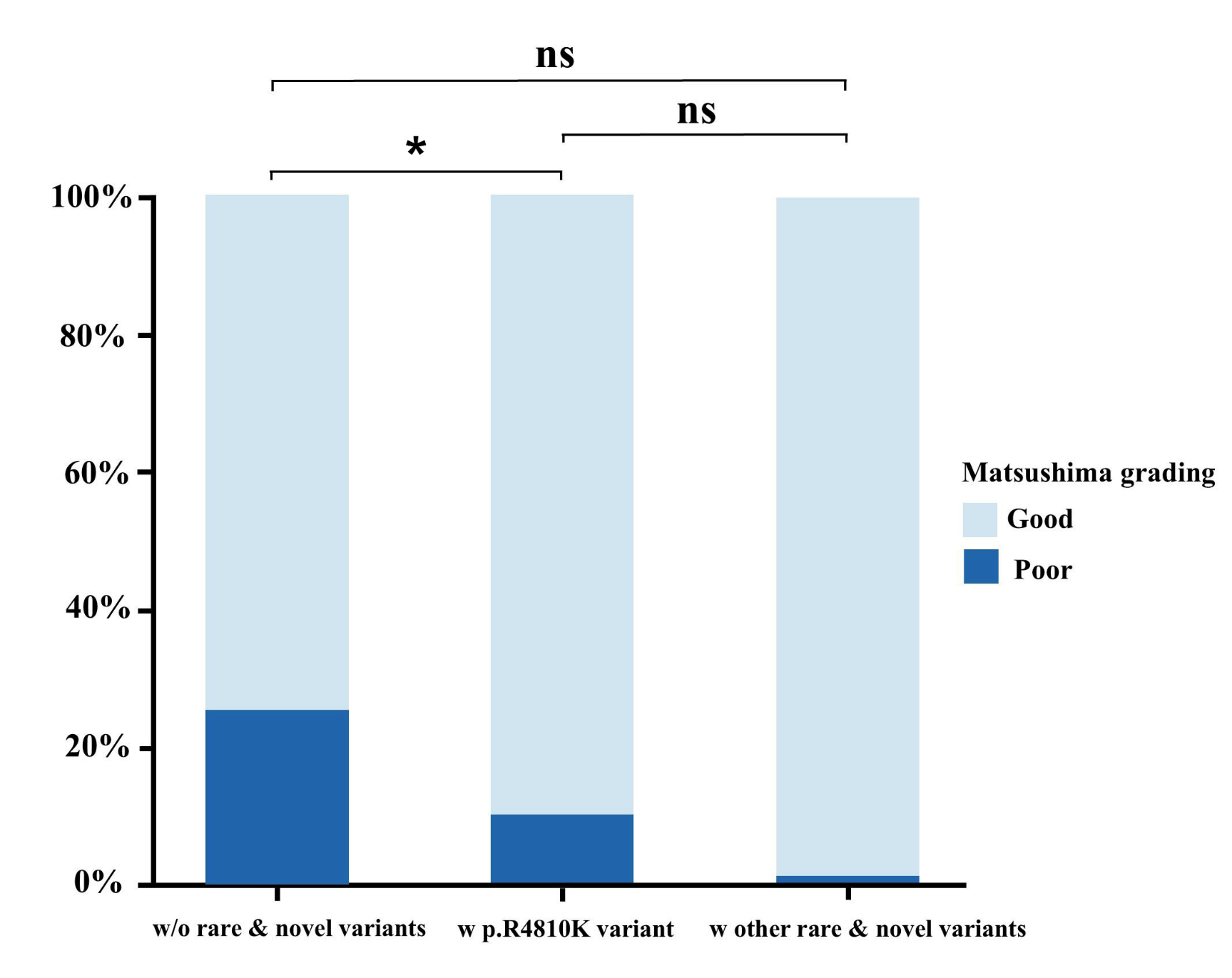
**

**Supplementary Figure 7.** Comparison of postoperative Matsushima grading across groups with different *RNF213* variants. The bar graph indicates that the p.R4810K variant group had a better postoperative Matsushima grade compared to the groups without other rare and novel variants. **p*<0.05. Figure is available in color online only.

**Supplementary References**

1 Liu W, Morito D, Takashima S, *et al.* Identification of RNF213 as a susceptibility gene for moyamoya disease and its possible role in vascular development. *PLoS One*. 2011;6:e22542

2 Wu Z, Jiang H, Zhang L, *et al*. Molecular analysis of RNF213 gene for moyamoya disease in the chinese han population. *PLoS One* 2012;7:e48179

3 Cecchi AC, Guo D, Ren Z, *et al*. RNF213 rare variants in an ethnically diverse population with moyamoya disease. *Stroke* 2014;45:3200-7

4 Moteki Y, Onda H, Kasuya H, *et al*. Systematic validation of RNF213 coding variants in japanese patients with moyamoya disease. *J Am Heart Assoc* 2015;4

5 Guey S, Kraemer M, Herve D, *et al*. Rare RNF213 variants in the c-terminal region encompassing the RING-finger domain are associated with moyamoya angiopathy in caucasians. *Eur J Hum Genet* 2017;25:995-1003

6 Jang MA, Chung JW, Yeon JY, *et al*. Frequency and significance of rare RNF213 variants in patients with adult moyamoya disease. *PLoS One* 2017;12:e0179689

7 Kobayashi H, Brozman M, Kyselova K, *et al*. RNF213 rare variants in slovakian and czech moyamoya disease patients. *PLoS One* 2016;11:e0164759

8 Zhang Q, Liu Y, Zhang D, *et al*. RNF213 as the major susceptibility gene for chinese patients with moyamoya disease and its clinical relevance. *J Neurosurg* 2017;126:1106-13

9 Hara S, Mukawa M, Akagawa H, *et al*. Absence of the RNF213 p.r4810k variant may indicate a severe form of pediatric moyamoya disease in japanese patients. *J Neurosurg Pediatr* 2022;29:48-56

10 Nomura S, Akagawa H, Yamaguchi K, *et al*. Difference in clinical phenotype, mutation position, and structural change of RNF213 rare variants between pediatric and adult japanese patients with moyamoya disease. *Transl Stroke Res* 2024;15:1142-53

11 Torazawa S, Miyawaki S, Imai H, *et al*. RNF213 p.arg4810lys wild type is associated with de novo hemorrhage in asymptomatic hemispheres with moyamoya disease. *Transl Stroke Res* 2024;15:729-38
